# Genome of tropical bed bug *Cimex hemipterus* (Cimicidae, Hemiptera) reveals tetraspanin expanded in bed bug ancestor

**DOI:** 10.1111/1744-7917.13388

**Published:** 2024-06-03

**Authors:** Sean Tsz Sum Law, Wenyan Nong, Chade Li, Tze Kiu Chong, Ho Yin Yip, Thomas Swale, Siu Wai Chiu, Roger Yat‐Nork Chung, Hon‐Ming Lam, Samuel Y. S. Wong, Hung Wong, Jerome H. L. Hui

**Affiliations:** ^1^ School of Life Sciences, Simon F.S. Li Marine Science Laboratory, State Key Laboratory of Agrobiotechnology, Institute of Environment, Energy and Sustainability The Chinese University of Hong Kong Hong Kong China; ^2^ Dovetail Genomics The United States of America; ^3^ School of Life Sciences The Chinese University of Hong Kong Hong Kong China; ^4^ School of Public Health and Primary Care, CUHK Institute of Health Equity The Chinese University of Hong Kong Hong Kong China; ^5^ School of Life Sciences, State Key Laboratory of Agrobiotechnology, Institute of Environment, Energy and Sustainability The Chinese University of Hong Kong Hong Kong China; ^6^ Department of Social Work, CUHK Institute of Health Equity, Institute of Environment, Energy and Sustainability The Chinese University of Hong Kong Hong Kong China

**Keywords:** bedbug, genome, microRNA, sesquiterpenoid, tetraspanin

## Abstract

*Cimex* species are ectoparasites that exclusively feed on warm‐blooded animals such as birds and mammals. Three cimicid species are known to be persistent pests for humans, including the tropical bed bug *Cimex hemipterus*, common bed bug *Cimex lectularius*, and Eastern bat bug *Leptocimex boueti*. To date, genomic information is restricted to the common bed bug *C. lectularius*, which limits understanding their biology and to provide controls of bed bug infestations. Here, a chromosomal‐level genome assembly of *C. hemipterus* (495 Mb [megabase pairs]) contained on 16 pseudochromosomes (scaffold N50 = 34 Mb), together with 9 messenger RNA and small RNA transcriptomes were obtained. In comparison between hemipteran genomes, we found that the tetraspanin superfamily was expanded in the *Cimex* ancestor. This study provides the first genome assembly for the tropical bed bug *C. hemipterus*, and offers an unprecedented opportunity to address questions relating to bed bug infestations, as well as genomic evolution to hemipterans more widely.

## Graphical abstract

Chromosomal level genome of tropical bedbug *Cimex hemipterus* provided in this study identifies the expansion of gene families in hemipterans, including tetraspanin family genes that are commonly expanded in bedbugs.

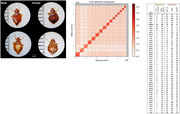

## Introduction

Cimicids in the Hemiptera are insects that feed on hosts’ blood. Despite the family Cimicidae contains 91 described species and can be divided into 6 subfamilies and 23 genera (Krinsky, 2019), most people refer bed bugs to the 3 better known species where humans serve as their hosts – tropical bed bug *Cimex hemipterus*, common bed bug *Cimex lectularius*, and Eastern bat bug *Leptocimex boueti*. People bitten by bed bugs could have reactions ranging from localized urticaria, bullous reactions, to anaphylaxis in rare cases (Thomas *et al.*, 2004). With globalization happening in the last half a century together with the easiness of cimicids transference, bed bugs are now considered as a worldwide resurging problem, with *C. hemipterus* and *C. lectularius* being the major species implicated in human infestations (Štefka *et al.*, 2022).

*C. hemipterus* and *C. lectularius*, similar to other hemipterans or true bugs, are hemimetabolous insects and their life cycle includes eggs, nymphal instars, and adults. All their instars and adults require taking blood meals from humans, although other mammals and birds can also be their hosts in the absence of humans. Female bed bugs can lay 5 eggs a day and eggs will further hatch into 1st instar nymphs in 4–12 d. A blood meal is essential for molting to the next developmental stage. Copulation or traumatic insemination happens between adults to fertilize eggs to complete the life cycle. As adults generally live 6–12 months and can survive for long periods of time without feeding, together with the occurrence of insecticide resistance, bed bug control and eradication is challenging and considered as a global problem (Romero *et al.*, 2007; Goddard & deShazo, 2009).

Obtaining the genome sequence of animals is useful for obtaining deeper understanding of their biology, as well as providing a valuable resource for varieties of follow‐up studies. In the case of bed bugs, understanding the underlying molecular mechanisms of their mating, feeding, and digestion on human blood could potentially be useful to make new measures for further control and eradication (Reinhardt & Siva‐Jothy, 2007). Nevertheless, to date, only the genome of common bed bug *C. lectularius* has been sequenced and analyzed (Benoit *et al.*, 2016; Rosenfeld *et al.*, 2016). Here, we aim to obtain a high‐quality genome and transcriptomic resources of the missing key infestation bed bug for humans, the tropical bed bug *C. hemipterus*, to shed light on the situation.

## Materials and methods

### Sample collection, genome sequencing, and assembly

Bed bugs were collected in different districts in Hong Kong, including Fanling, Kwai Hing, Kwun Tong, Shatin, Tsing Yi, and Tsuen Wan. Genomic DNA (gDNA) was extracted with QIAamp DNA Mini Kit (Qiagen), and sent to BGI Hong Kong Company Limited for single‐tube long fragment read (stLFR) sequencing on DNBSEQ G400 to obtain 2 × 100 bp paired‐end reads (Peters *et al.*, 2015; Wang *et al.*, 2019). Dovetail Omni‐C library was further prepared as described (Lieberman‐Aiden *et al.*, 2009), and sequenced on Illumina HiSeq X to give 2 × 150 bp paired‐end reads. The stLFR reads were used for *de novo* assembly according to the stLFR pipeline (https://github.com/BGI‐Qingdao/stlfr2supernova_pipeline) (Weisenfeld *et al.*, 2017; Wang *et al.*, 2019), with default parameters. The pseudohap style output dedupe contigs and Dovetail Omni‐C library reads were used as input data for HiRise, a software pipeline specifically designed to use proximity ligation data to scaffold genome assemblies (Putnam *et al.*, 2016). Sequences from the Dovetail Omni‐C library were aligned to the draft input assembly using Burrows‐Wheeler Aligner (BWA) (https://github.com/lh3/bwa). The separation of Dovetail Omni‐C read pairs mapped within the draft scaffolds was analyzed using HiRise to build a likelihood model for the genomic distance between read pairs. The model was used to identify and break putative misjoins, score potential joins and make joins above a threshold. Estimation of genome size, repeat content, and heterozygosity were carried out by GenomeScope2 with *K*‐mer at 31 (Vurture *et al.*, 2017), which was then compared with 2 *C. lectularius* genomes in the National Center for Biotechnology Information (NCBI) (Accession numbers GCA_000648675.3 and GCA_001460545.1). Syntenic analysis between the X, Y chromosome of the Heteroptera genomes (*Aelia acuminata*, GCA_911387785.2; *Acanthosoma haemorrhoidale* GCA_930367205.1; *Aradus depressus* GCA_963662175.1) and the *C. hemipterus* genome was run using SyMAP (Soderlund *et al.*, 2011). Details of the sequencing data can be found in Table S1.

### Transcriptome sequencing

Total RNA was extracted from adult samples using the mirVana miRNA Isolation Kit (Ambion) according to the manufacturer's protocol. The quality of extracted total RNA was checked using a Nanodrop spectrophotometer (Thermo Scientific), gel electrophoresis, and an Agilent 2100 Bioanalyzer (Agilent RNA 6000 Nano Kit). Qualified samples were sent to Novogene (HK) Company Limited for library construction and sequencing. PolyA‐selected RNA sequencing libraries were prepared using the TruSeq RNA Sample Prep Kit v2. Insert sizes and library concentrations of the final libraries were examined using an Agilent 2100 Bioanalyzer instrument (Agilent DNA 1000 Reagents). Similarly, isolated small RNA was submitted to Novogene (HK) Company Limited for HiSeq small RNA library construction and 50 bp single end (SE) sequencing. Details of the sequencing data can be found in Table S1. Raw sequencing reads from 5 transcriptomes were preprocessed with quality trimmed by trimmomatic (v0.39 with parameters “ILLUMINACLIP:TruSeq3‐PE.fa:2:30:10 SLIDINGWINDOW:4:5 LEADING:5 TRAILING:5 MINLEN:25”) (Bolger *et al.*, 2014) and contamination removed by Kraken2 (database version k2_standard_20210517) (Wood *et al.*, 2019). The processed reads were used for the following gene model prediction.

### Gene model prediction

The gene model was predicted as previously described (Li *et al.*, 2020). Briefly, gene models were trained and predicted using funannotate (v1.8.9, https://github.com/nextgenusfs/funannotate) with the parameters “–repeats2evm –protein_evidence uniprot_sprot.fasta –genemark_mode ET –busco_seed_species fly –optimize_augustus –busco_db insecta –organism other –max_intronlen 350000”. Gene models from several prediction sources, including GeneMark, Augustus high‐quality predictions (HiQ), pasa, Augustus, GlimmerHM, and snap, were passed to Evidence Modeler (EVM) and used to generate gene model annotation files and applied in Program to Assemble Spliced Alignments (PASA) to update EVM consensus predictions, add untranslated region (UTR) annotations and models for alternatively spliced isoforms. Protein‐coding gene models were then blasted (blastp) to the NCBI nr and Swiss‐Prot databases using diamond (v0.9.24), specifying the following parameters: –more‐sensitive –e‐value 1e‐3, and mapped with transcriptome reads using HISAT2 (version 2.1.0) (Kim *et al.*, 2019). Gene models with no homology to known proteins in the nr and Swiss‐Prot databases, and without messenger RNA (mRNA) support, were removed from the final version. Gene model expression profiles were generated from different individuals by extracting transcript per million (TPM) values from mapped reads using StringTie (PERTEA *et al.*, 2015).

### Gene orthology and gene family evolution analysis

Orthologous groups were inferred from the longest representative protein sequences of 29 hemipterans including *C. hemipterus* downloaded from NCBI (https://www.ncbi.nlm.nih.gov/datasets/genome/GCF_000648675.2/) and 21 arthropod taxa using OrthoFinder v2.5.2 (Emms & Kelly, 2019) (Table S5). The default settings were used and the multiple sequence alignments option “‐MSA” was activated to generate a maximum likelihood of species tree, which was further inferred for divergence times with r8s (Sanderson, 2003) and TimeTree web database (http://timetree.org/). The orthologous groups and species tree were used as input in CAFE5 analysis (Mendes *et al.*, 2020). Candidates of expanded gene families were inspected by protein family search with HMMER (version 3.3.1; cut‐off *E*‐value < 10^−5^) (Eddy, 2011), from which each gene candidate was validated with Conserved Domain Database (CDD) *via* RPS‐Basic Local Alignment Search Tool (BLAST) (Yang *et al.*, 2020). Subsequently, gene families of interest were investigated for sequence alignment using MAFFT v7.455 (Katoh & Standley, 2013), followed by phylogenetic analysis with FastTree (Price *et al.*, 2010) and visualization in evolview v3 (Subramanian *et al.*, 2019). For the classification of tetraspanin families, reference sequences from Huang *et al.* (2005) were used. The pairwise sequence identity was computed by Clustal Omega (v1.2.4, Sievers *et al.*, 2011). Microsynteny between *C. hemipterus* and *C. lectularius* was identified with MCScanX (Wang *et al.*, 2012) followed by visualization with *gggenomes* (Hackl *et al.*, 2021). DeepTMHMM (Hallgren *et al.*, 2022) was employed to annotate the transmembrane regions of tetraspanin.

### Gene family annotation and gene tree building

*Drosophila melanogaster* Hox and ParaHox genes were retrieved from HomeoDB (Zhong & Holland, 2011) to search for Hox and ParaHox genes in both *C. hemipterus* and *C. lectularius* using tBLASTn with threshold of *E*‐value of <10^−3^. Reference homeobox gene sequences were retrieved from HomeoDB and a published study (Qu *et al.*, 2020). For hormonal and anticoagulation genes annotation, amino sequences were first obtained from Kyoto Encyclopedia of Genes and Genomes (KEGG) or the NCBI gene database. The obtained sequences were then used to retrieve the corresponding genes from *C. hemipterus* genome using tBLASTn with threshold of *E*‐value of <10^−3^. All annotated genes were further proceeded to phylogenetic analyses using the neighbor‐joining method with MEGA7 (Kumar *et al.*, 2016) and maximum likelihood method using IQTREE (version 2.2.0.3) (Schrempf *et al.*, 2016). The phylogenetic tree was then visualized with iTOL (Letunic & Bork, 2021). Expression of the annotated hormonal genes were visualized by heatmap illustration using TBtools (Version No.1.112) (Chen *et al.*, 2020).

### MicroRNA annotations

Quality of small RNA sequencing reads were checked using FastQC (Andrews, 2010), and reads between 18 and 27 bp in length were then mapped to the genome using mapper.pl. MicroRNAs were predicted using miRDeep2.pl from miRDeep2 (Mackowiak, 2011). Expression profiles and quantification were generated by the miRDeep2 script quantifier.pl with 0 mismatches in read‐to‐predecessor mapping and other default parameters. The raw count table was used for differential expression analysis by Trinity's run_DE_analysis.pl and analyse_diff_expr.pl scripts using the edgeR method, where at least 2 replicates had calculated counts per million (CPM) values ≥ 1, and cut‐offs were set at *P = *0.05 (false discovery rate) and 2‐fold changes. Predicted microRNAs in *C. hemipterus* were manually checked by using BLASTn in miRBase (Kozomara *et al.*, 2019) with default settings, as well as aligning them with reference microRNA hairpin sequences from MirGeneDB (Fromm *et al.*, 2022). The genomic locations of all annotated microRNAs were visualized with Tbtools (Chen *et al.*, 2020), and the distance between neighboring members of a microRNA cluster is set to 10 kb (kilobase pairs) (Kozomara *et al.*, 2019). The microRNA of *C. lectularius* was retrieved from InsectBase 2.0 (Mei *et al.*, 2022). The presence of nonannotated microRNAs hairpin of *C. lectularius* was also checked by using BLASTn to the genomes with microRNA hairpin of *C. hemipterus* as reference.

### Repeat annotation

Transposable elements (TEs) were annotated as previously described (Baril and Hayward, 2022) using the automated Earl Grey TE annotation pipeline (version 1.2, https://github.com/TobyBaril/EarlGrey, Baril *et al.*, 2024). In brief, this pipeline first involved the identification of known TEs from the lepidopteran subset of Dfam (release 3.4) and RepBase (release 20 181 026) (Hubley *et al.*, 2016). An automated “BLAST, Extract, Extend” process was then used to identify *de novo* TEs and extend consensus boundaries (Platt *et al.*, 2016). Redundant sequences were removed from the consensus library before the genome assembly was annotated with the combination of known and *de novo* TE libraries. The annotations were then processed to remove overlaps and to defragment the annotations prior to the final TE quantification.

## Results and Discussion

### A high‐quality genome of *C. hemipterus*

**Fig. 1:**
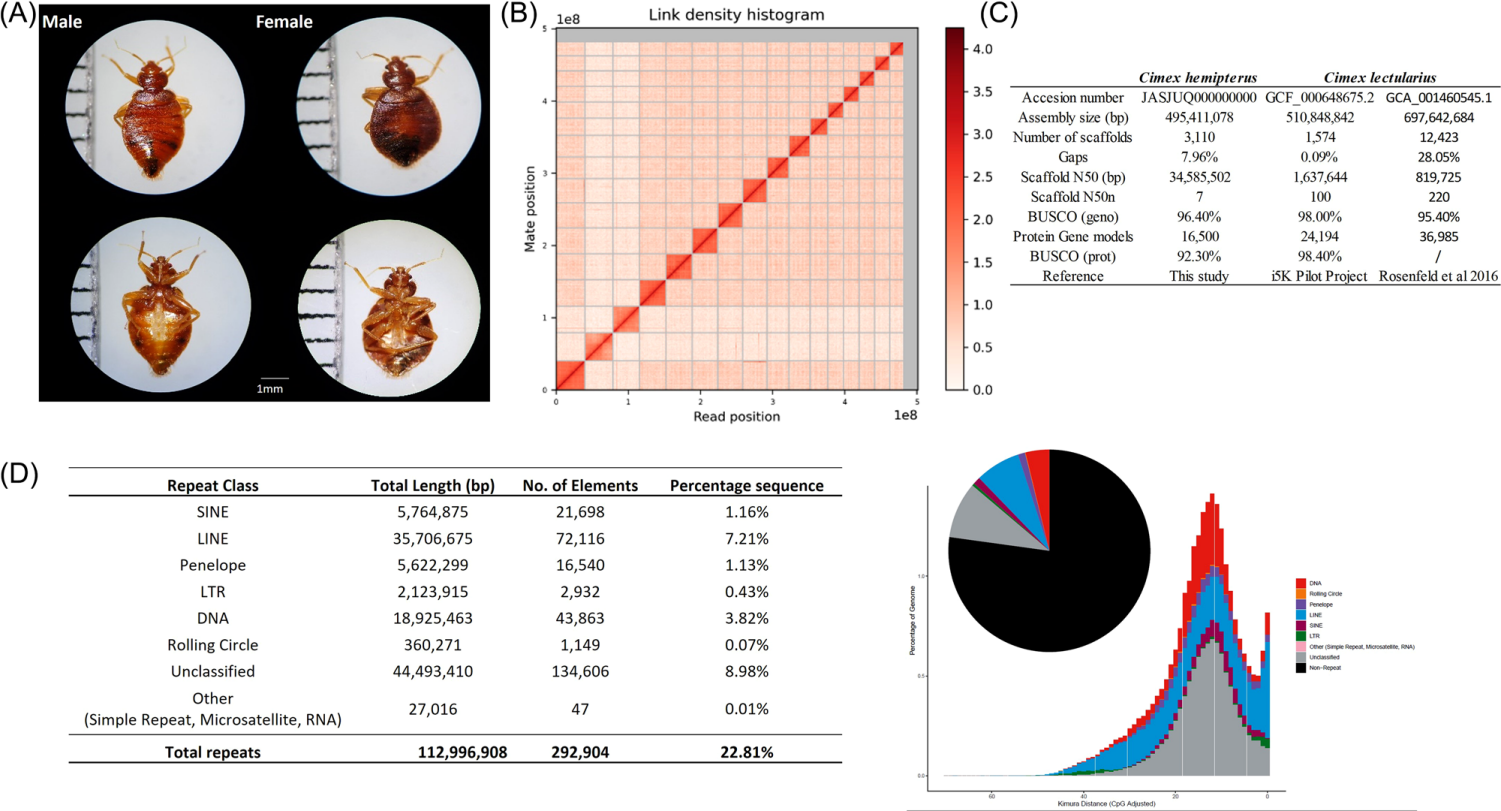
(A) Photographs of male and female of tropical bed bug *Cimex hemipterus*. (B) Hi‐C interaction of 16 linkage groups. (C) Statistics of *C. hemipterus* and *C. lectularius* genome assemblies. (D) Transposable elements in *C. hemipterus*.

Here, we present a high‐quality chromosome‐level genome assembly of *C. hemipterus* (2*n* = 32), with 97.25% of the genome sequence contained on 16 pseudomolecules (∼495.4 Mb [megabase pairs], scaffold N50 = 34.6 Mb) (Fig. 1B,C; Table S2), from which macrosynteny revealed Chr 2 and Chr 3 are X_1_ and X_2_ choromosomes that correspond to the male karyotype (2*n* = 28 + X_1_X_2_Y) (Sadílek *et al.*, 2019) (Fig. S1), where the Y chromosome was not assembled in the genome. The size of the assembled genome is comparable with the estimated size of the genome (484.2 Mb) and the 2 *C. lecutlarius* genomes (553 and 517 Mb for GCF_000648675.2 and GCA_001460545.1, respectively) (Fig. S2). A total of 33 254 predicted gene models were annotated using the transcriptomes generated in this study, including 16 500 protein‐coding genes and 16 754 transfer RNA genes, and the genome completeness estimated by Benchmarking Universal Single‐Copy Orthologs (BUSCO) analyses was 96.40% (Fig. 1C and Table S3). We have also identified a total of 22.81% repeat contents in the *C. hemipterus* genome (Fig. 1D), with many of these newly discovered repetitive elements relatively understudied in current databases (unclassified: 8.98%). Among the remaining identifiable repeats, long interspersed nuclear element (LINE) elements and DNA transposons are the most abundant (LINEs: 7.21%, DNA transposons: 3.82%), whereas short interspersed nuclear elements (SINEs), long terminal repeat (LTR) elements, and rolling circle elements are only present in low proportions (LTR elements: 0.43%, rolling circle elements: 0.07%, SINEs: 1.16%) (Fig. 1D), which is similar to the bed bug *C. lectularius* (Rosenfeld *et al.*, 2016). Our genome in this study provides the first chromosome‐level genome in the Cimicidae (Table S4).

### Anticoagulant proteins and gene family evolution in the Cimex lineage

Hematophagous animals such as bed bugs have the ability to secrete anticoagulants to prevent blood clotting and triggering other immune responses of the hosts in order to obtain blood meals. Similar to *C. lectularius*, we can also identify genes involved in the production of different anticoagulant proteins (Figs. S3–S5; Tables S12 and S13).

**Fig. 2:**
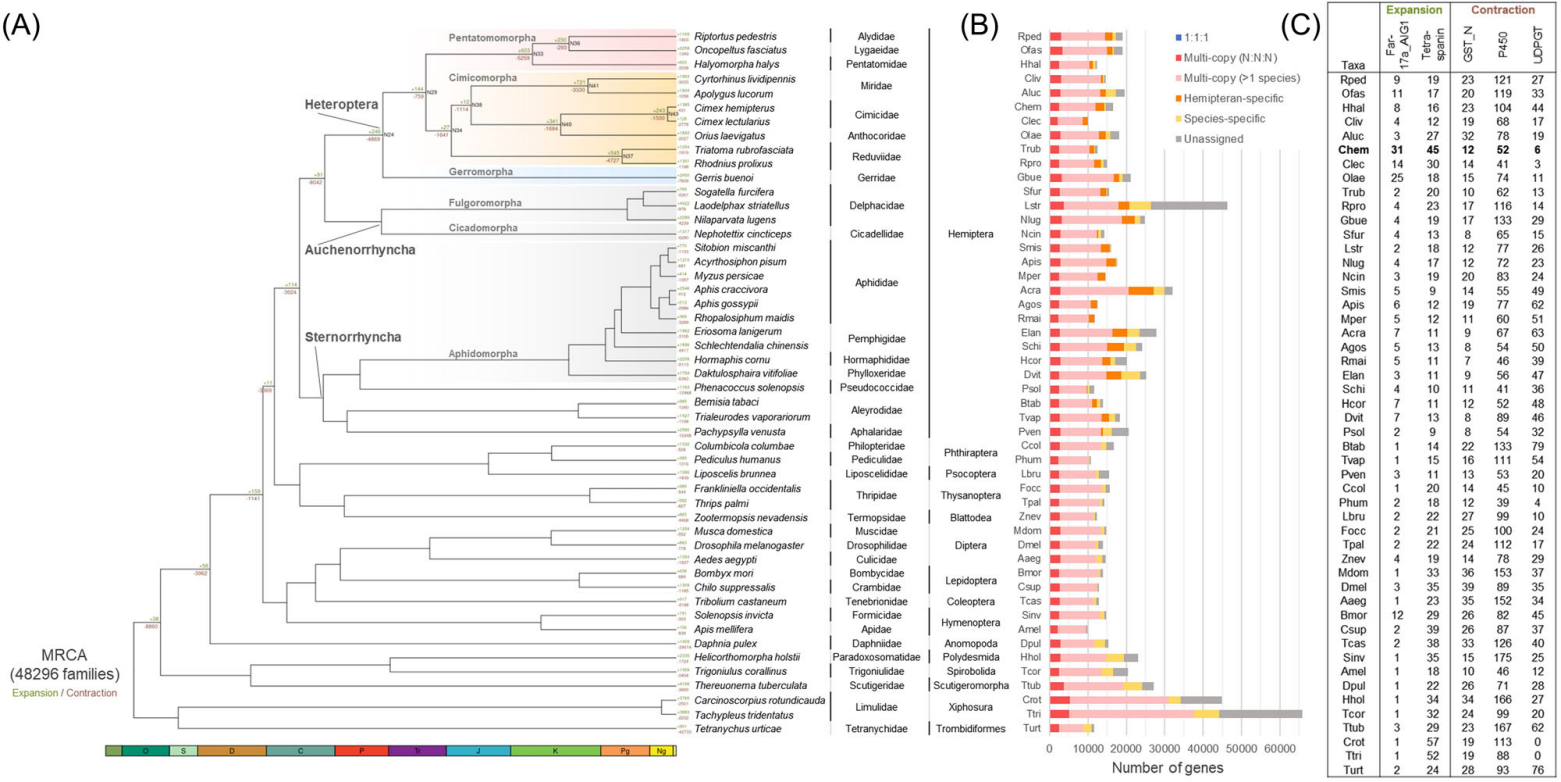
(A) Summary of gene family expansion and contraction in *Cimex* and other reference animals. MRCA, most recent common ancestor. (B) Number and distribution of genes assigned to different orthologous groups. (C) Gene count table with expanded or contracted protein family domains.

To understand what other adaptations could have arisen in the *Cimex* ancestor, genome‐wide gene gain and loss analyses across insects with the inclusion of this new genomic resource were carried out (Fig. 2A, B). We have found that 2 transmembrane protein families, FAR‐17a/AIG1‐like protein and tetraspanin (also known as TM4SF), were significantly expanded in both *Cimex* genomes (Fig. 2C). A larger number of gene copies of FAR‐17a/AIG1‐like proteins was detected in *C. hemipterus* (*n* = 31) when compared to *C. lectularius* (*n* = 14). The difference was made by tandem gene duplicates in *C. hemipterus*, including a cluster of 9 FAR‐17a/AIG1‐like protein‐coding genes on chromosome 16 (Fig. S6A). Although all annotated FAR‐17a/AIG1‐like proteins were expressed in *C. hemipterus* (Fig. S2B), little is known about their function in insects as FAR‐17a/AIG1‐like proteins were mainly characterized in vertebrate models as an androgen‐responsive gene and a role player in cancer‐related processes (Huang *et al.*, 2021). On the other hand, in addition to known insect tetraspanin orthologous families (Huang *et al.*, 2005), the expansion in tetraspanin was provided by a newly identified family termed “N‐3”, which was also found in hemipteran *Orius laevigatus* (Figs. S7 and S8). Moreover, not only some tetraspanins in the N‐3 cluster contained a novel “CCS” cysteine pattern of large extracellular domain (EC2) instead of the conserved “CCG” signature, but most N‐3 tetraspanins were expressed in males only (Fig. S8). It is noteworthy that tandem gene duplications of N‐3 tetraspanins were also identified in both *Cimex* species, in which larger gene clusters were observed in *C. hemipterus* and led to a larger gene number of tetraspanins than in *C. lectularius* (Fig. S8A). In animals, tetraspanins are well‐known membrane‐spanning proteins that function as molecular scaffolds contributing to development, reproduction, and immunity (Termini & Gillette, 2017). In *Drosophila* fruit flies, species‐specific tetraspanin *Sunglasses* (DmTsp42Ej) is required for retina development, and for flies with extended light exposure, but having reduced *Sunglasses* expression resulted in the inability to regulate rhodopsin (Xu *et al.*, 2004). Given that bed bugs are nocturnal and are able to detect colors and objects at very low background light conditions (Singh *et al.*, 2015), whether *Cimex* tetraspanins could potentially be involved in visual acuity would be an interesting story to test. Understanding the functions of these *Cimex‐*specific tetraspanins and whether they can be used as candidates for combating bed bugs, similar to the development of potential therapeutic targets in human diseases, warrants further investigation.

### Developmental and hormonal genes in *C. hemipterus*

**Fig. 3:**
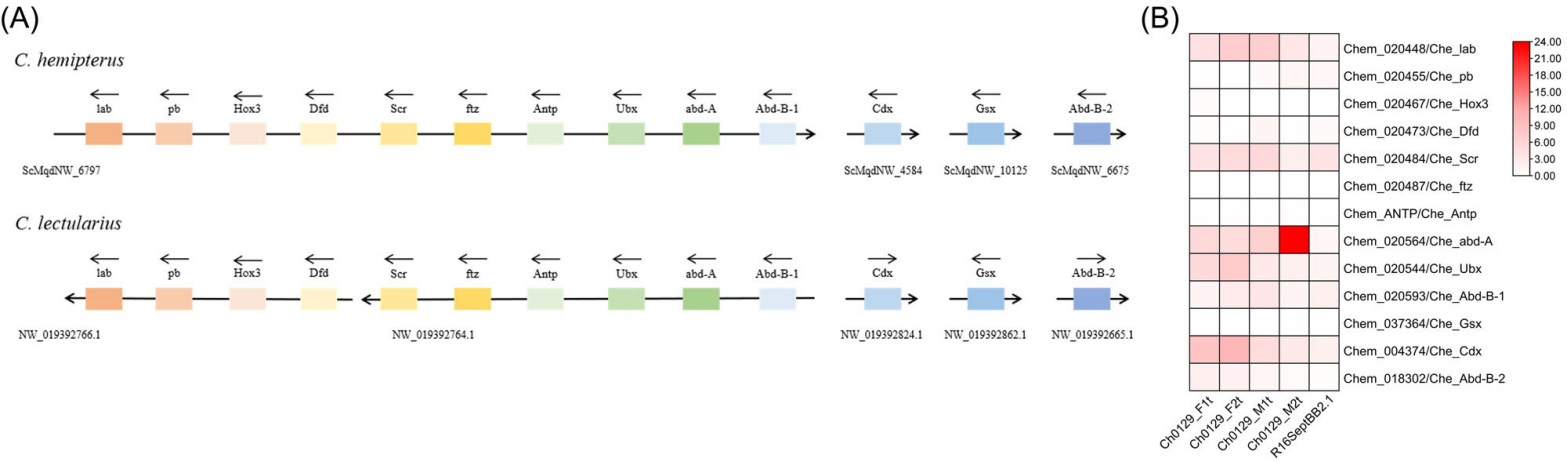
(A) Genomic locations of Hox and ParaHox genes in *Cimex hemipterus* and *C. lectularius*. (B) Expression of Hox and ParaHox genes in *C. hemipterus*.

Homeobox genes such as Hox and ParaHox genes are transcription factors involved in regulating animal development, and their genomic organization can help understanding how animals evolved (*e.g*., Nong *et al.*, 2020). A total of 13 Hox and ParaHox genes were identified in each of *C. hemipterus* and *C. lectularius* genomes (Fig. 3A, Table S6 and Fig. S9). In *C. hemipterus* genome, the majority of Hox genes are clustered on the same scaffold with exception of 1 posterior Hox gene, while the 2 ParaHox genes are located on 2 different scaffolds. The situation contrasts with that of the *C. lectularius*, where the majority of Hox genes are located on 2 different scaffolds, presumably due to lower scaffold N50 (Fig. 3A). It is worth noting that the posterior Hox genes were both located on a different scaffold from the majority of Hox genes in both *Cimex* genomes, suggesting the translocation event could be dated back to the *Cimex* last common ancestor. The Hox and ParaHox genes expressed largely similarly between sexes (Fig. 3B and Table S7).

**Fig. 4:**
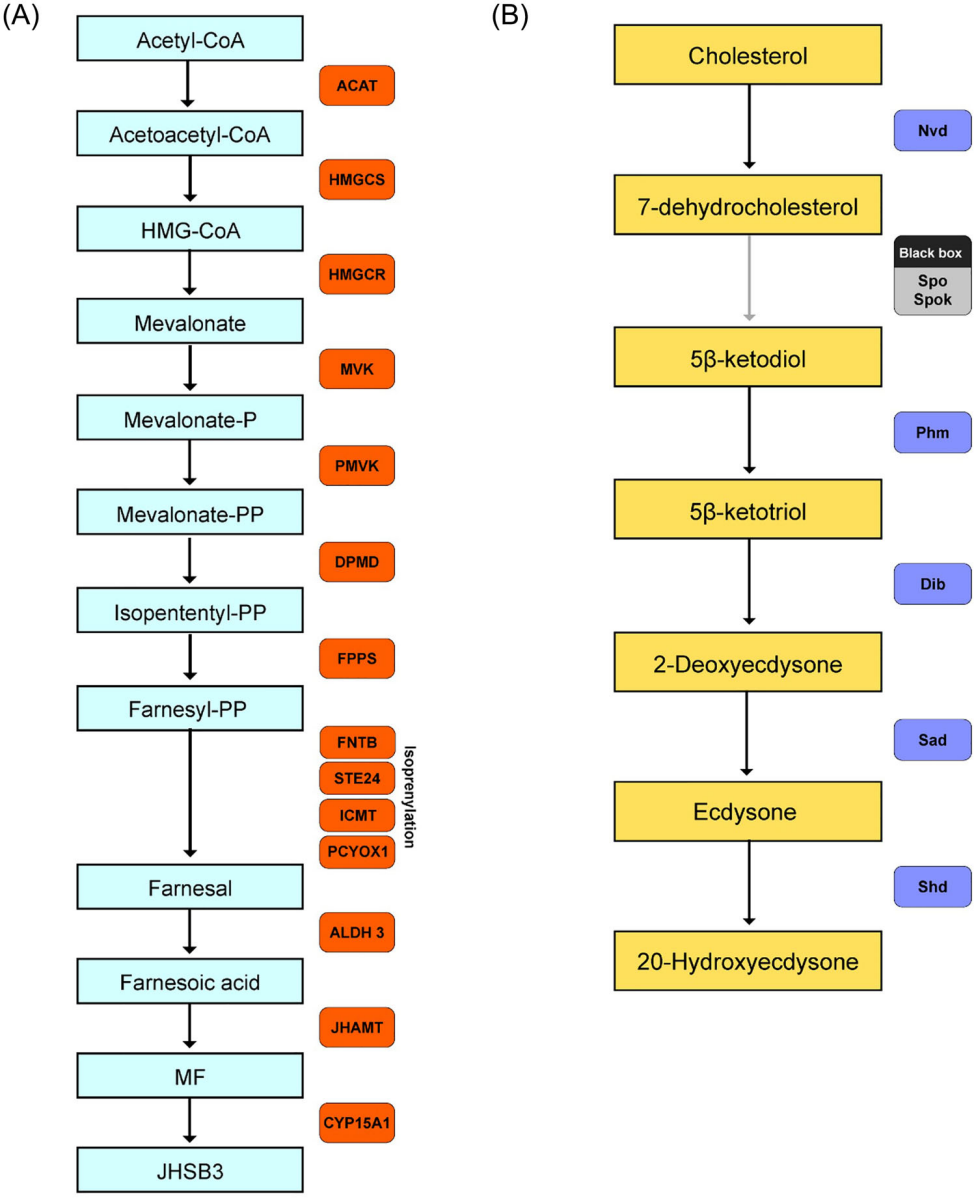
Sesquiterpenoid and ecdysteroid pathway genes in tropical bed bug *Cimex hemipterus*. (A) Schematic diagram of sesquiterpenoid biosynthetic pathway genes identified in *C. hemipterus*. *ACAT*, acetyl‐CoA acetyltransferase; *ALDH 3*, aldehyde dehydrogenase 3; *DPMD*, diphosphomevalonate decarboxylase; *FNTB*, protein farnesyl transferase; *FPPS*, farnesyl diphosphate synthase; *JHAMT*, juvenile hormone acid *O*‐methyltransferase; *HMGCR*, 3‐hydroxy‐3‐methylglutaryl‐CoA reductase; *HMGCS*, hydroxymethylglutaryl‐CoA synthase; *ICMT*, protein‐S‐isoprenylcysteine *O*‐methyltransferase; *MVK*, mevalonate kinase; *PCYOX1*, prenylcysteine oxidase; *PMVK*, phosphomevalonate kinase; *STE24*, endopeptidase. (B) Sesquiterpenoid pathway genes identified in *C. hemipterus*. *Nvd*, Neverland; *Spo*, Spook; *Spok*, Spookier; *Phm*, Phantom; *Dib*, disembodied; *Sad*, Shadow; *Shd*, Shade.

Sesquiterpenoid hormones such as juvenile hormones control development and reproduction in insects, and are potential targets for controlling insect growth and numbers. In the *C. hemipterus* genome, genes involved in the mevalonate (MVA) pathway, including *ACAT*, *HMGCS*, *HMGCR*, *MVK*, *PMVK*, *DPMD*, and *FPPS*, were identified (Fig. 4A; Table S8 and Figs. S10–S16), suggesting *C. hemipterus* starts sesquiterpenoid production by the MVA pathway (Tobe & Bendena 1999; Qu *et al.*, 2018; Tsang *et al.*, 2020; So *et al.*, 2022). However, *FPPP* and *FOHSDR* which code for enzymes to convert farnesyl‐PP to farnesol and farnesal respectively as in many other arthropods were not detected in the *C. hemipterus* genome (Baker *et al.*, 1983; Mayoral *et al.*, 2009; Nyati *et al.*, 2013; Satvaveanthan *et al.*, 2021). Instead, gene members involved in the isoprenylation pathway, namely *FNTB*, *STE24*, *ICMT*, and *PCYOX1*, were detected, indicating that *C. hemipterus* may produce farnesal through the isoprenylation pathway (Zhang & Casey, 1996) (Figs. S17–S20). In addition, we could identify orthologs of *ALDH III*, *JHAMT*, and *CYP15A1* (Fig. 4A; Table S8 and Figure S21–S23), suggesting FA, MF, and JHSB3 were being synthesized in *C. hemipterus*, which is consistent with other hemipterans (Kotaki *et al.*, 2009; Rivera‐Perez *et al.*, 2013; Tsang *et al.*, 2020; Villalobos‐Sambucaro *et al.*, 2020; So *et al.*, 2022). Comparing the gene expression level between the 2 sexes, we found there was a higher gene expression level of *HMGCR* and lower gene expression level of a *STE24* copy in the 2 female samples (Ch0129_F1t, Ch0129_F2t) than the 2 male samples (Ch0129_Mt1, Ch0129_Mt2). However, the gene expression level of sesquiterpenoid genes of another bed bug species *C. lectularius* do not show any sex‐biased expression (Table S9 and Fig. S24).

Other than sesquiterpenoids, ecdysteroids, are another group of important hormones that regulate growth (such as molting and metamorphosis) and sexual maturation of insects (Cheong *et al.*, 2015). In the *C. hemipterus* genome, *Spo*/*Spok*, *Nvd*, *Phm*, *Dib*, *Sad*, and *Shd* could all be identified (Fig. 4B, Table S10 and Fig. S25), suggesting *C. hemipterus* shared a similar ecdysteroid biosynthesis pathway to other insects, in which cholesterol is converted to 7‐dehydrocholesterol (7dC) and then to 5*β*‐ketodiol (Namiki *et al.*, 2005; Ono *et al.*, 2006; Warren *et al.*, 2009; Niwa *et al.*, 2010; Ou *et al.*, 2011; Niwa & Niwa, 2014), followed by further hydroxylation where 5*β*‐ketotriol, 2‐deoxyecdysone, ecdysone, and 20‐hydroxyecdysone are synthesized, respectively (Niwa & Niwa, 2014). Comparing the gene expression level between the 2 sexes, except for *Nvd*, the expression level of all annotated ecdysteroid genes were found to be higher in the females than males. Similar to sesquiterpenoid, the gene expression level of ecdysteroid genes of another bed bug species *C. lectularius* do not show any sex‐biased expression (Table S11 and Fig. S26).

### MicroRNAs in *C. hemipterus* and other hemipterans

**Fig. 5:**
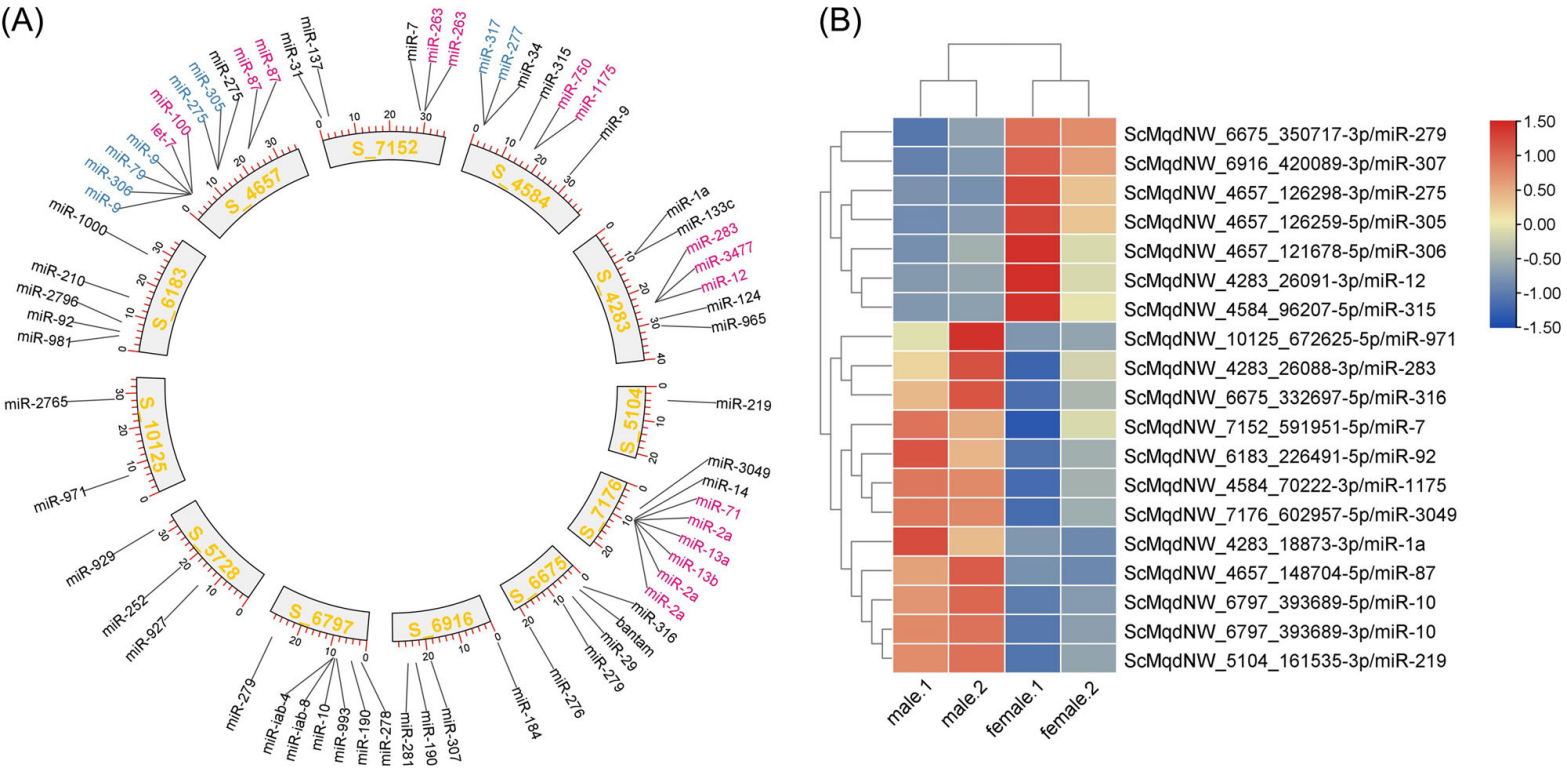
(A) Genomic locations of microRNAs in tropical bed bug *Cimex hemipterus*. MicroRNA clusters are alternatively colored in blue and red; (B) microRNA differential expression genes.

MicroRNAs are important post‐transcriptional regulators in insects, but the microRNAs in *C. hemipterus* remain unexplored to date. Utilizing the small RNA transcriptomes generated in this study, a total of 65 microRNAs could be confidently identified on 12 scaffolds (Fig. 5A and Table S14). Similar to other animals, some of these microRNAs were arranged in conserved genomic clusters, including miR‐283/3477/12, miR‐317/277, miR‐750/1175, miR‐9/306/79/9, miR‐275/305, miR‐87/87, let‐7/miR‐100, miR‐263/263, and miR‐71/2/13/13/2/2 (Fig. 5A) (Marco *et al.*, 2013; Ikeda *et al.*, 2015; Chang *et al.*, 2016). By comparison of the microRNA expression levels between the 2 sexes in *C. hemipterus*, we also discovered that miR‐12, miR‐275, miR‐279, miR‐305, miR‐306, miR‐307, and miR‐315 were expressed in a higher level in females than males, while miR‐10, miR‐1175, miR‐1a, miR‐219, miR‐283, miR‐3049, miR‐316, miR‐7, miR‐87, miR‐92, and miR‐971 were in a higher expression level in males than females (Fig. 5B, Tables S15 and S16). How these microRNAs control the feeding behaviors between sexes remains to be elucidated.

## Conclusion

This study presents the first high‐quality genome assembly for the tropical bed bug, which is scientifically important and has considerable medical relevance. Our work provides gene, TE, and microRNA annotations, and their detailed evolutionary analyses. More generally, our high‐quality *C. hemipterus* genome, transcriptomic resources, and population data provide a useful reference point for further understanding of the biology, ecology, evolution, and infestation of insect pests.

## Disclosure

The authors declare no conflict of interests.

## Supporting information


**Fig. S1** Synteny between the X, Y chromosome of the Heteroptera genomes (*Aelia acuminata*, GCA_911387785.2; *Acanthosoma haemorrhoidale* GCA_930367205.1; *Aradus depressus* GCA_963662175.1) downloaded from NCBI and the genome of this study.
**Fig. S2** GenomeScope reports on the heterozygosity, repeat content and size of the genome with kmer 31.
**Fig. S3** Phylogenetic gene tree of apyrase genes identified in *C. hemipterus*.
**Fig. S4** Phylogenetic gene tree of salivary nitrophorin genes identified in *C. hemipterus*.
**Fig. S5** Phylogenetic gene tree of Kazal‐type thrombin inhibitor genes identified in *C. hemipterus*.
**Fig. S6** Genomic location and expression profile of Far‐17/AIG1 protein coding genes.
**Fig. S7** Classification of tetraspanin in *C. hemipterus* and other outgroup taxa.
**Fig. S8** Summary of tetraspanin in *C. hemipterus*.
**Fig. S9** Phylogenetic gene tree of Hox and ParaHox identified in *C. hemipterus* and *C. lectularius*.
**Fig. S10** Phylogenetic gene tree of ACAT identified in *C. hemipterus*.
**Fig. S11** Phylogenetic gene tree of HMGCS identified in *C. hemipterus*.
**Fig. S12** Phylogenetic gene tree of HMGCR identified in *C. hemipterus*.
**Fig. S13** Phylogenetic gene tree of MVK identified in *C. hemipterus*.
**Fig. S14** Phylogenetic gene tree of PMVK identified in *C. hemipterus*.
**Fig. S15** Phylogenetic gene tree of DPMD identified in *C. hemipterus*.
**Fig. S16** Phylogenetic gene tree of FPPS identified in *C. hemipterus*.
**Fig. S17** Phylogenetic gene tree of FNTB identified in *C. hemipterus*.
**Fig. S18** Phylogenetic gene tree of STE24 identified in *C. hemipterus*.
**Fig. S19** Phylogenetic gene tree of ICMT identified in *C. hemipterus*.
**Fig. S20** Phylogenetic gene tree of PCYOX1 identified in *C. hemipterus*.
**Fig. S21** Phylogenetic gene tree of ALDH3 identified in *C. hemipterus*.
**Fig. S22** Phylogenetic gene tree of JHAMT identified in *C. hemipterus*.
**Fig. S23** Phylogenetic gene tree of CYP15A1 identified in *C. hemipterus*.
**Fig. S24** Gene expression level of sesquiterpenoid genes in *C. hemipterus* and *C. lectularius*.
**Fig. S25** Phylogentic gene tree of ecdysteroid genes, Nvd, Spo/Spok, Phm, Dib, Sad, and Shd, identified in *C. hemipterus*.
**Fig. S26** Gene expression level of ecdysteroid genes in *C. hemipterus* and *C. lectularius*.

**Table S1** Sequencing information of gDNA, mRNA, sRNA and population.
**Table S2** Statistics top 16 pseudomolecules of *C. hemipterus* genome.
**Table S3** Summary of BUSCO statistics.
**Table S4** Statistics on hemipteran genomes in the NCBI.
**Table S5** Statistics on comparative genomes.
**Table S6** Hox and ParaHox genes in *C. lectularius* and *C. hemipterus*.
**Table S7** Expression of Hox and ParaHox genes in *C. hemipterus*.
**Table S8** Sesquiterpenoid genes of *C. hemipterus*.
**Table S9** Expression of sesquiterpenoid genes in *C. hemipterus* and *C. lectularius*.
**Table S10** Ecdysteroid genes of *C. hemipterus*.
**Table S11** Expression of ecdysteroid genes in *C. hemipterus* and *C. lectularius*.
**Table S12** Anticoagulation genes of *C. hemipterus*.
**Table S13** Expression of anticoagulation genes in *C. hemipterus*.
**Table S14** MicroRNA annotation.
**Table S15** MicroRNA expression table.
**Table S16** MicroRNA differential expression genes.

## Data Availability

The final chromosome assembly was submitted to NCBI Assembly under accession number JASJUQ000000000 in NCBI. The raw reads generated in this study have been deposited into the NCBI database under the BioProject accession PRJNA713496; the genome annotation files were deposited in the Figshare (https://figshare.com/s/d9956fa56189ac8938c0).
