# Supplementary material for: Genome of tropical bed bug Cimex hemipterus (Cimicidae, Hemiptera) reveals tetraspanin expanded in bed bug ancestor
Source: Insect Sci. 2024 Jun 3;32(1):42–54. doi: 10.1111/1744-7917.13388 (PMC11824892; doi:10.1111/1744-7917.13388)
Supplement: Supplementary file 1 — Fig. S1 Synteny between the X, Y chromosome of the Heteroptera genomes (Aelia acuminata, GCA_911387785.2; Acanthosoma haemorrhoidale GCA_930367205.1; Aradus depressus GCA_963662175.1) downloaded from NCBI and the genome of this study. Fig. S2 GenomeScope reports on the heterozygosity, repeat content and size of the genome with kmer 31. Fig. S3 Phylogenetic gene tree of apyrase genes identified in C. hemipterus. Fig. S4 Phylogenetic gene tree of salivary nitrophorin genes identified in C. hemipterus. Fig. S5 Phylogenetic gene tree of Kazal‐type thrombin inhibitor genes identified in C. hemipterus. Fig. S6 Genomic location and expression profile of Far‐17/AIG1 protein coding genes. Fig. S7 Classification of tetraspanin in C. hemipterus and other outgroup taxa. Fig. S8 Summary of tetraspanin in C. hemipterus. Fig. S9 Phylogenetic gene tree of Hox and ParaHox identified in C. hemipterus and C. lectularius. Fig. S10 Phylogenetic gene tree of ACAT identified in C. hemipterus. Fig. S11 Phylogenetic gene tree of HMGCS identified in C. hemipterus. Fig. S12 Phylogenetic gene tree of HMGCR identified in C. hemipterus. Fig. S13 Phylogenetic gene tree of MVK identified in C. hemipterus. Fig. S14 Phylogenetic gene tree of PMVK identified in C. hemipterus. Fig. S15 Phylogenetic gene tree of DPMD identified in C. hemipterus. Fig. S16 Phylogenetic gene tree of FPPS identified in C. hemipterus. Fig. S17 Phylogenetic gene tree of FNTB identified in C. hemipterus. Fig. S18 Phylogenetic gene tree of STE24 identified in C. hemipterus. Fig. S19 Phylogenetic gene tree of ICMT identified in C. hemipterus. Fig. S20 Phylogenetic gene tree of PCYOX1 identified in C. hemipterus. Fig. S21 Phylogenetic gene tree of ALDH3 identified in C. hemipterus. Fig. S22 Phylogenetic gene tree of JHAMT identified in C. hemipterus. Fig. S23 Phylogenetic gene tree of CYP15A1 identified in C. hemipterus. Fig. S24 Gene expression level of sesquiterpenoid genes in C. hemipterus and C. lectularius. Fig. S25 Phyloge [file INS-32-42-s001.pptx]

## Slide 1
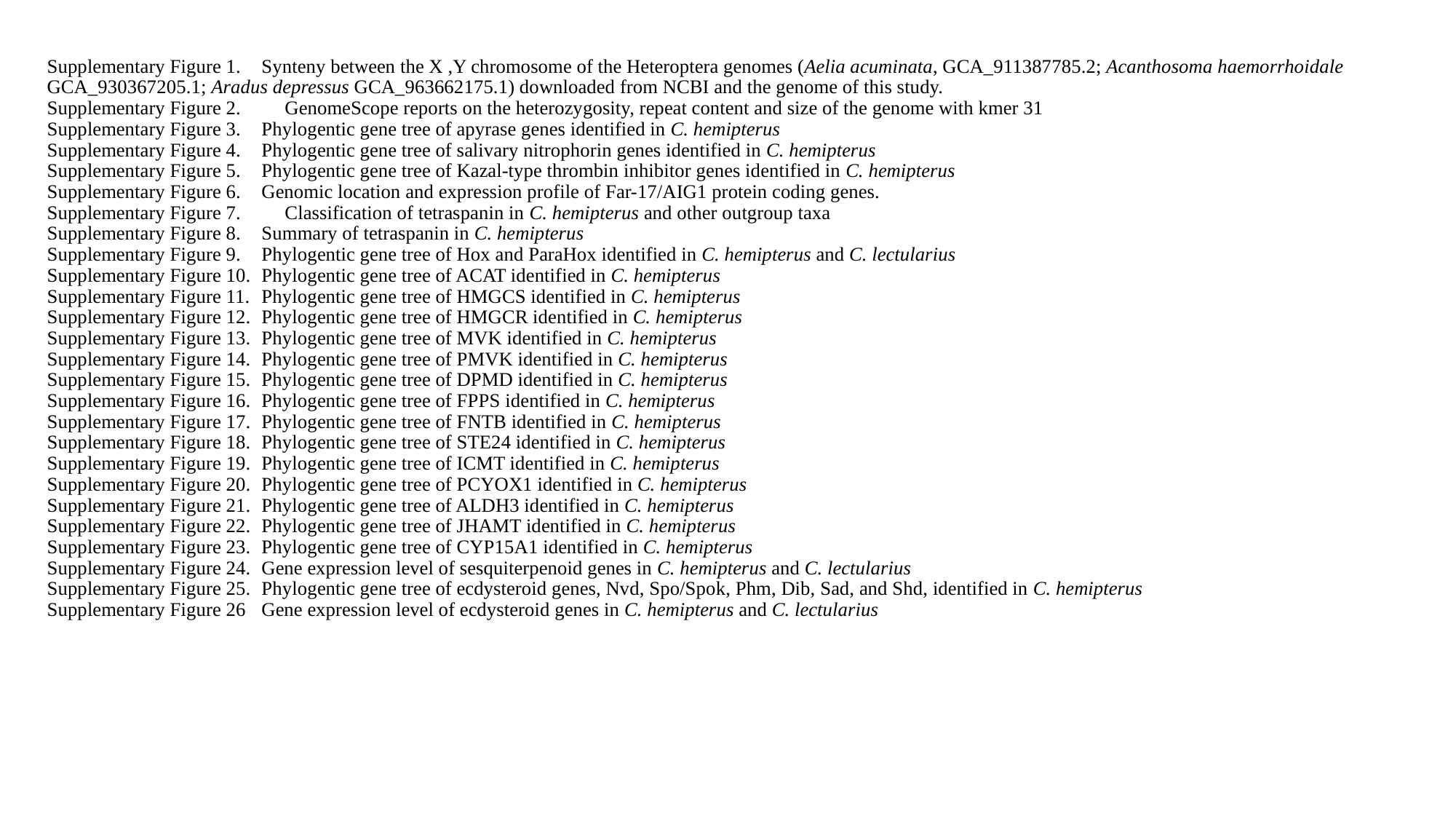

# Supplementary Figure 1.	 Synteny between the X ,Y chromosome of the Heteroptera genomes (Aelia acuminata, GCA_911387785.2; Acanthosoma haemorrhoidale GCA_930367205.1; Aradus depressus GCA_963662175.1) downloaded from NCBI and the genome of this study. Supplementary Figure 2. GenomeScope reports on the heterozygosity, repeat content and size of the genome with kmer 31 Supplementary Figure 3.	 Phylogentic gene tree of apyrase genes identified in C. hemipterus Supplementary Figure 4.	 Phylogentic gene tree of salivary nitrophorin genes identified in C. hemipterus Supplementary Figure 5.	 Phylogentic gene tree of Kazal-type thrombin inhibitor genes identified in C. hemipterus Supplementary Figure 6.	 Genomic location and expression profile of Far-17/AIG1 protein coding genes. Supplementary Figure 7. Classification of tetraspanin in C. hemipterus and other outgroup taxa Supplementary Figure 8. 	 Summary of tetraspanin in C. hemipterus Supplementary Figure 9.	 Phylogentic gene tree of Hox and ParaHox identified in C. hemipterus and C. lectularius Supplementary Figure 10.	 Phylogentic gene tree of ACAT identified in C. hemipterus Supplementary Figure 11.	 Phylogentic gene tree of HMGCS identified in C. hemipterus Supplementary Figure 12.	 Phylogentic gene tree of HMGCR identified in C. hemipterus Supplementary Figure 13.	 Phylogentic gene tree of MVK identified in C. hemipterus Supplementary Figure 14.	 Phylogentic gene tree of PMVK identified in C. hemipterus Supplementary Figure 15.	 Phylogentic gene tree of DPMD identified in C. hemipterus Supplementary Figure 16.	 Phylogentic gene tree of FPPS identified in C. hemipterus Supplementary Figure 17.	 Phylogentic gene tree of FNTB identified in C. hemipterus Supplementary Figure 18.	 Phylogentic gene tree of STE24 identified in C. hemipterus Supplementary Figure 19.	 Phylogentic gene tree of ICMT identified in C. hemipterus Supplementary Figure 20.	 Phylogentic gene tree of PCYOX1 identified in C. hemipterus Supplementary Figure 21.	 Phylogentic gene tree of ALDH3 identified in C. hemipterus Supplementary Figure 22.	 Phylogentic gene tree of JHAMT identified in C. hemipterus Supplementary Figure 23.	 Phylogentic gene tree of CYP15A1 identified in C. hemipterus Supplementary Figure 24. 	 Gene expression level of sesquiterpenoid genes in C. hemipterus and C. lectularius Supplementary Figure 25.	 Phylogentic gene tree of ecdysteroid genes, Nvd, Spo/Spok, Phm, Dib, Sad, and Shd, identified in C. hemipterus Supplementary Figure 26	 Gene expression level of ecdysteroid genes in C. hemipterus and C. lectularius

## Slide 2
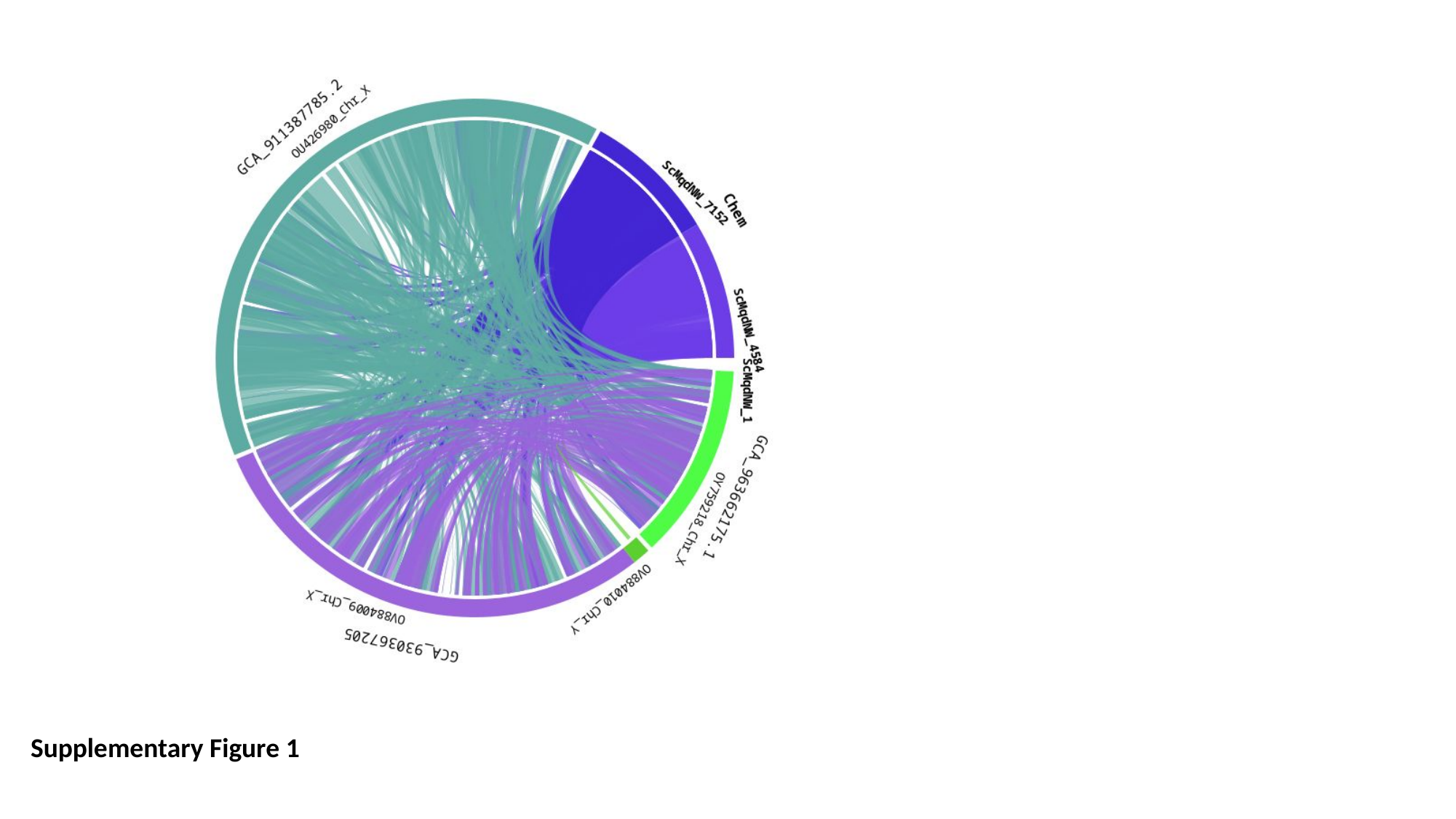

Supplementary Figure 1

## Slide 3
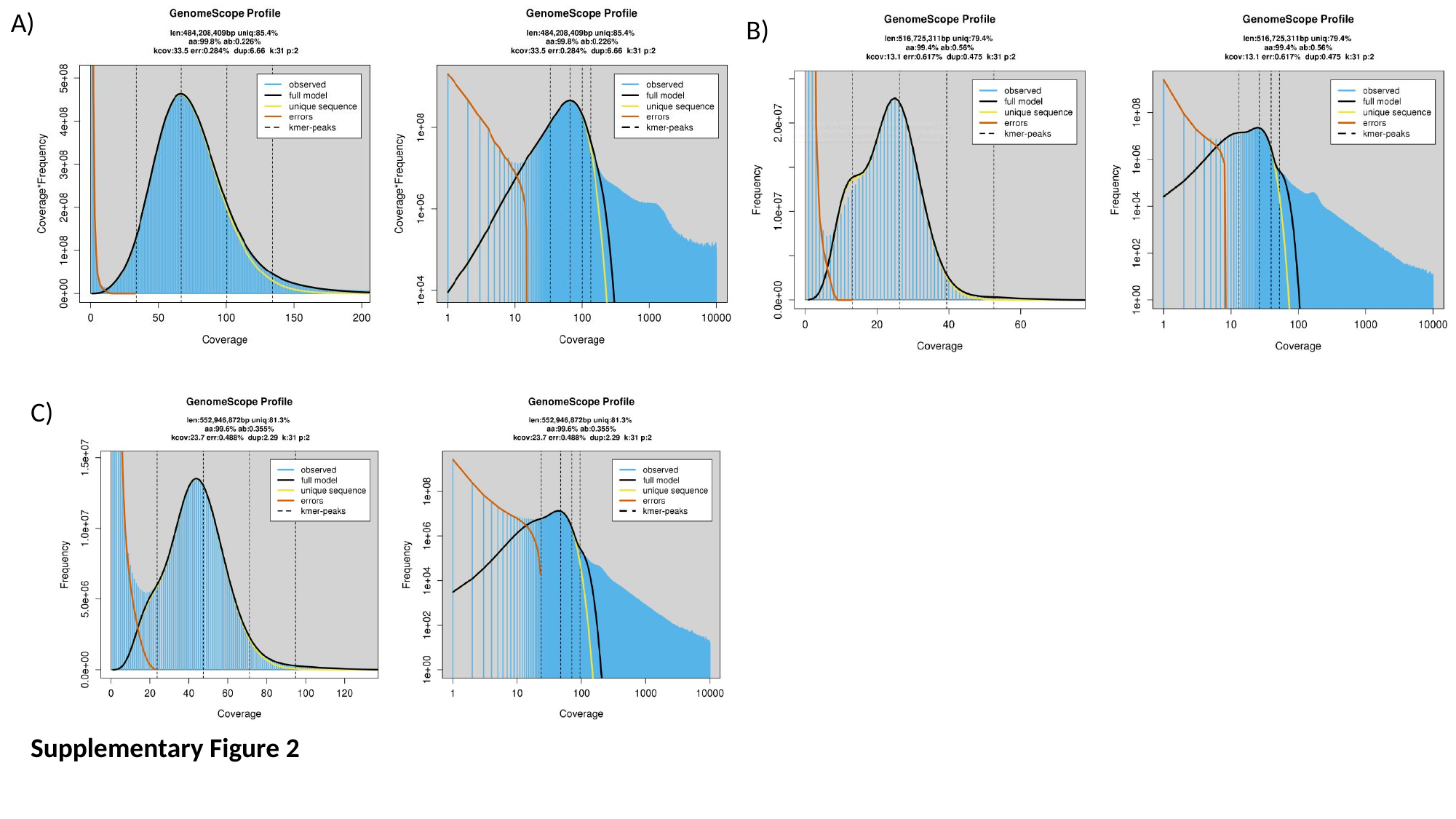

A)
B)
C)
Supplementary Figure 2

## Slide 4
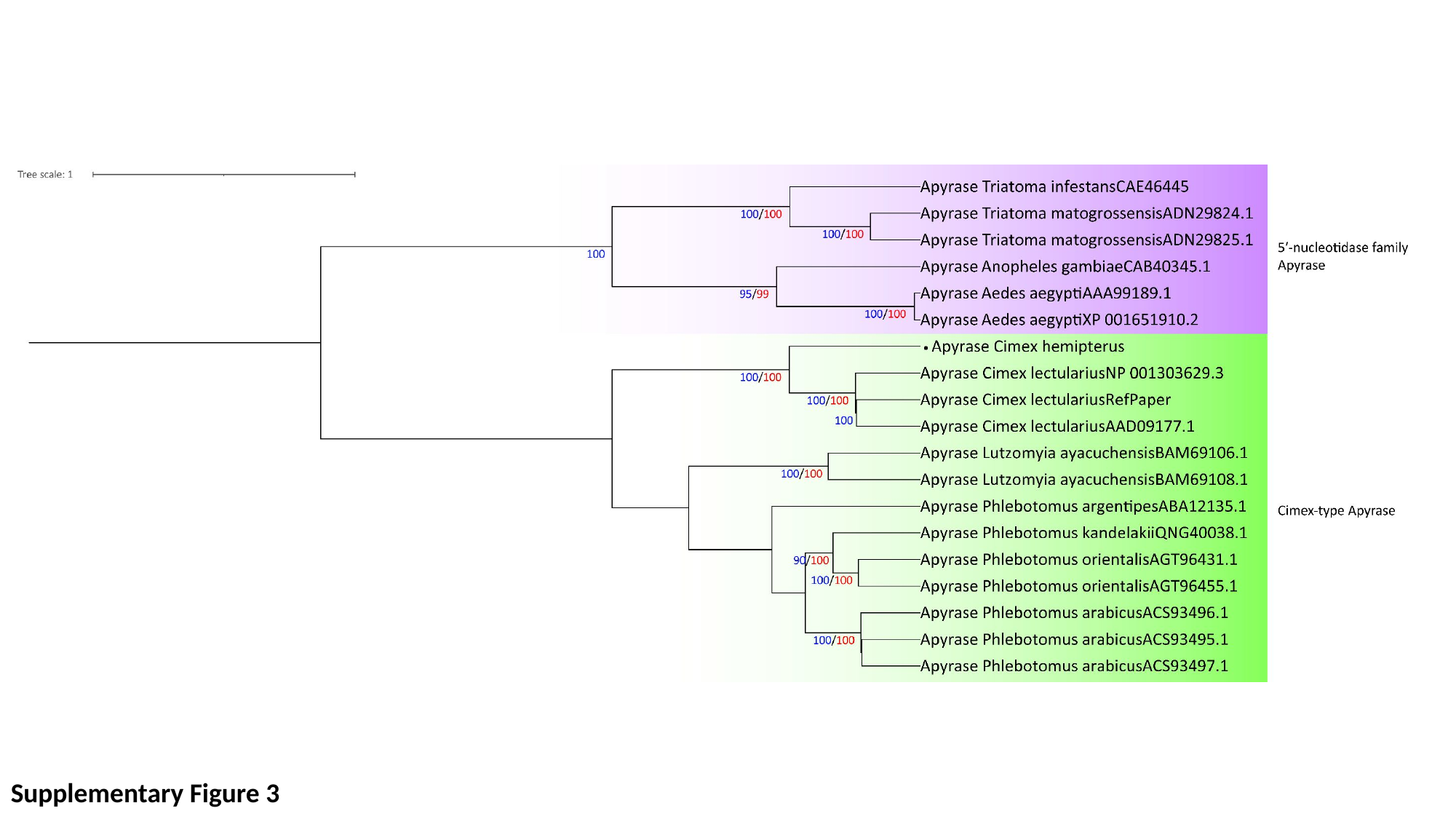

Supplementary Figure 3

## Slide 5
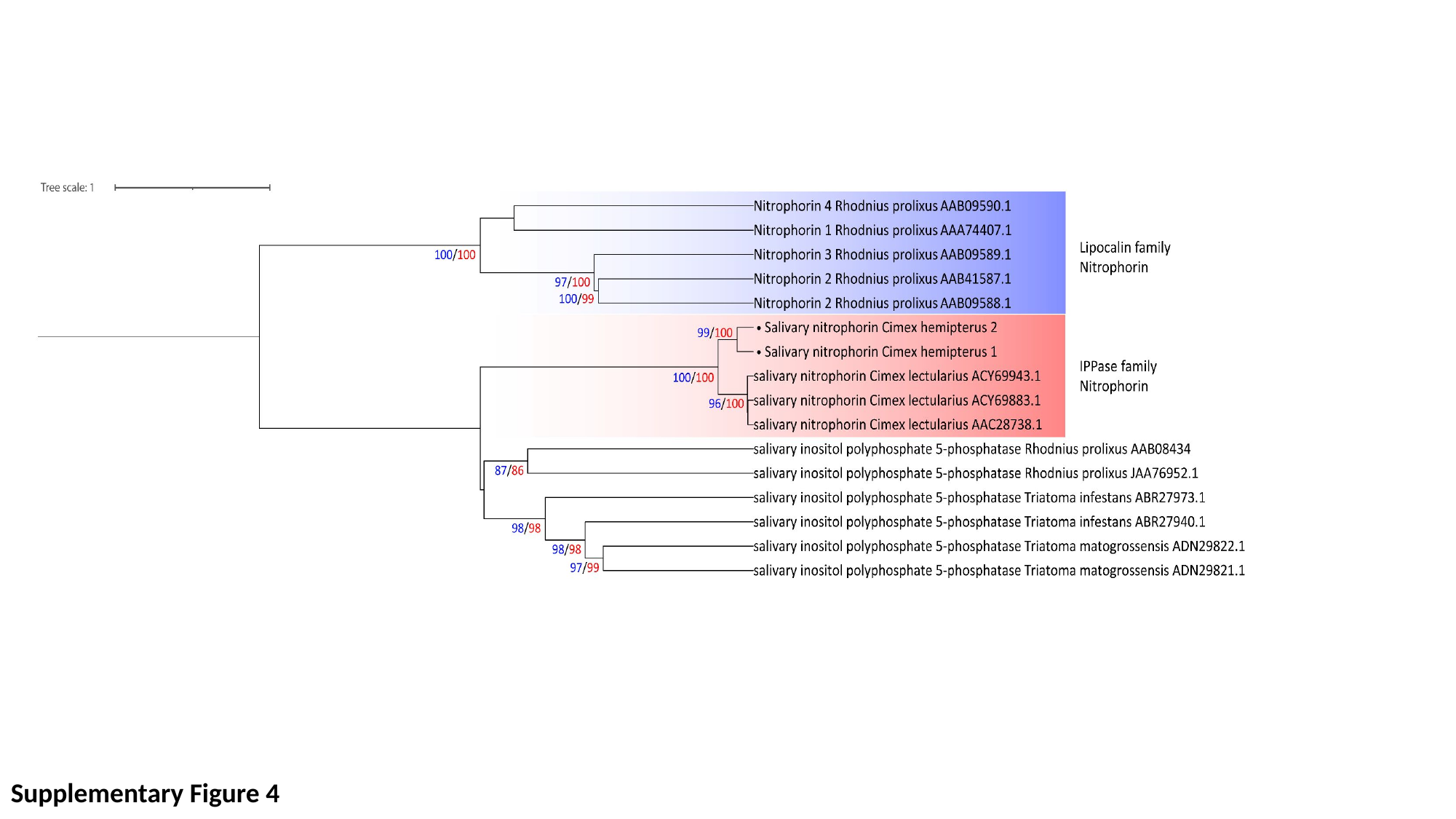

Supplementary Figure 4

## Slide 6
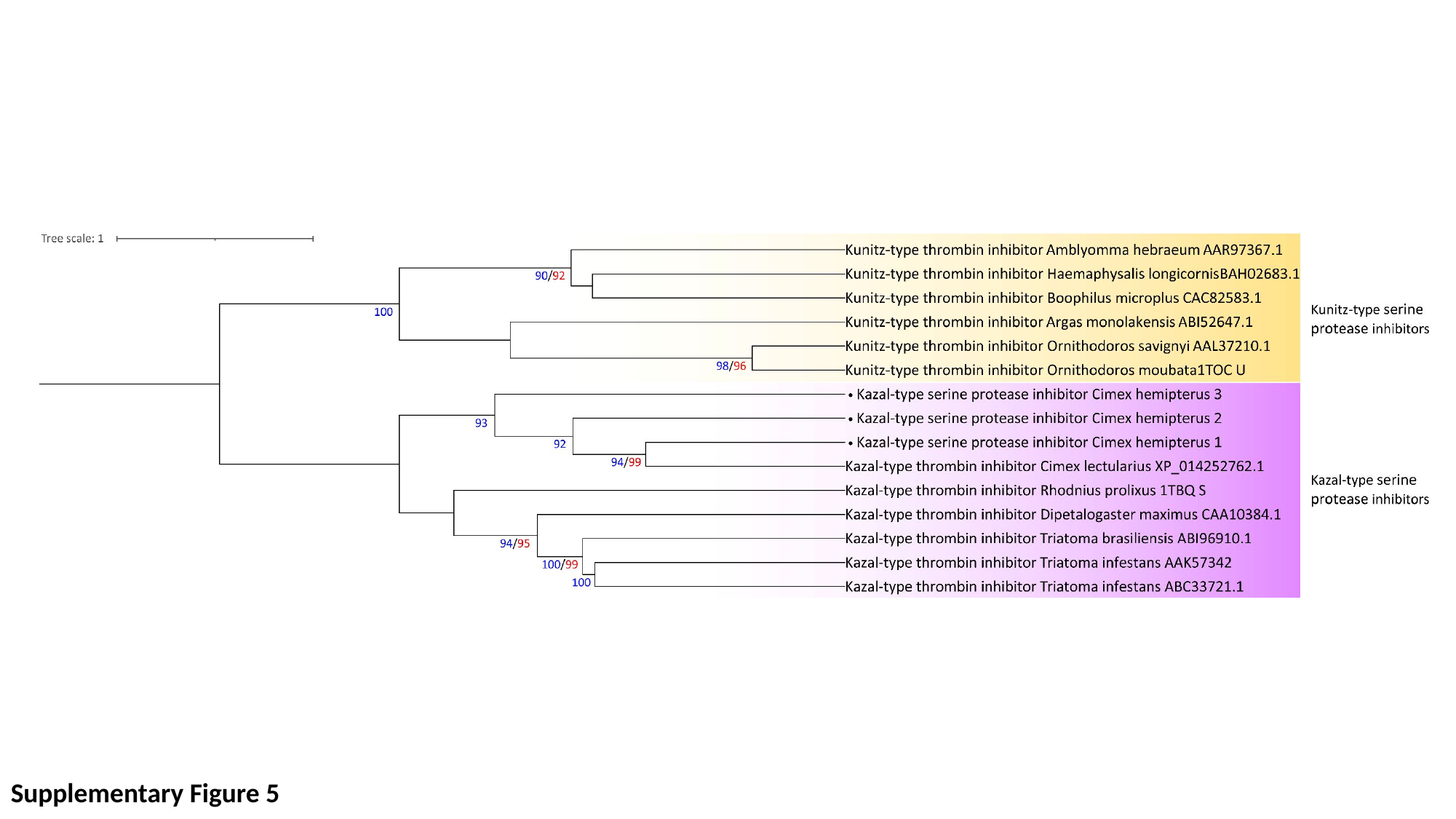

Supplementary Figure 5

## Slide 7
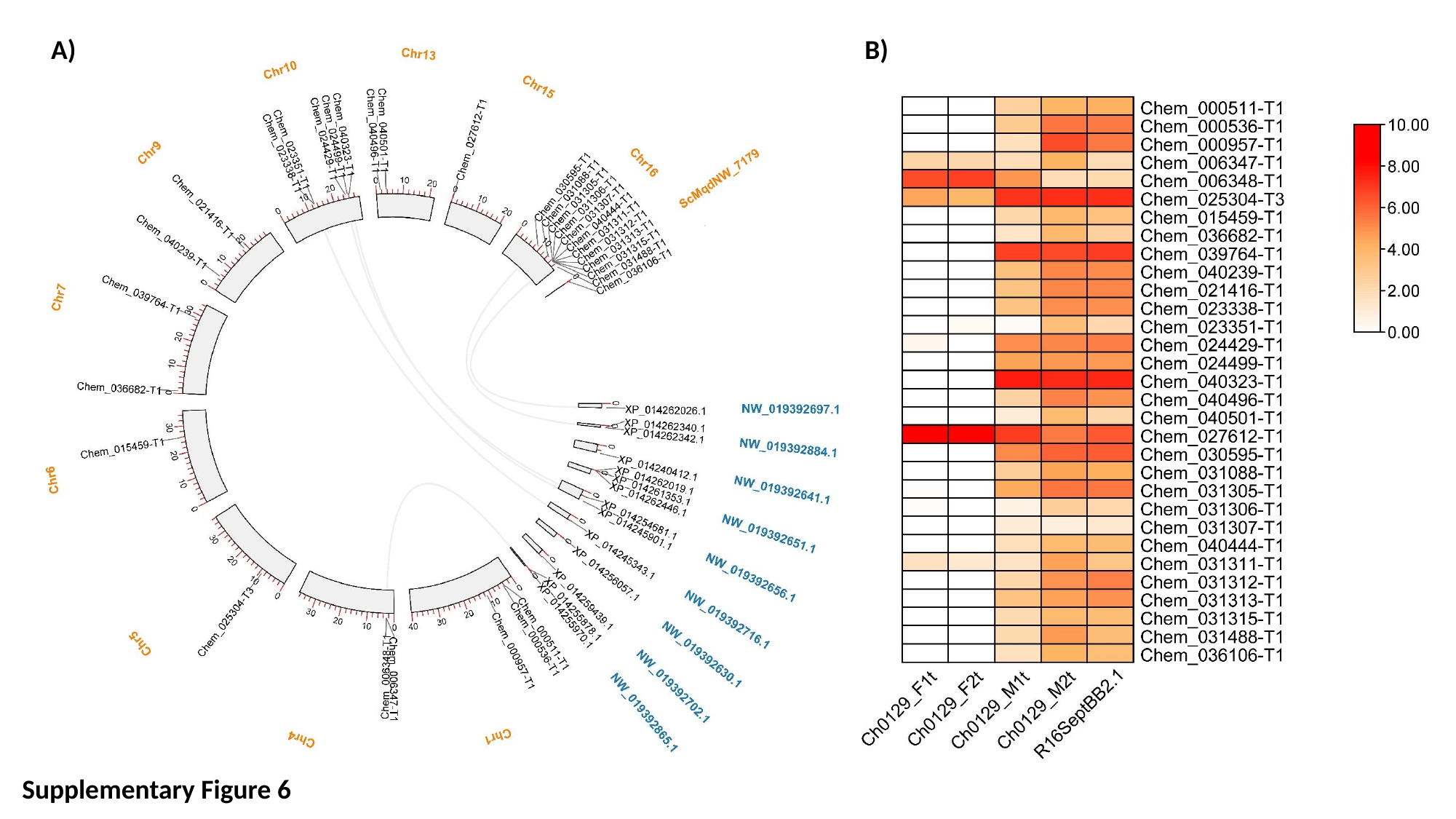

A)
B)
Supplementary Figure 6

## Slide 8
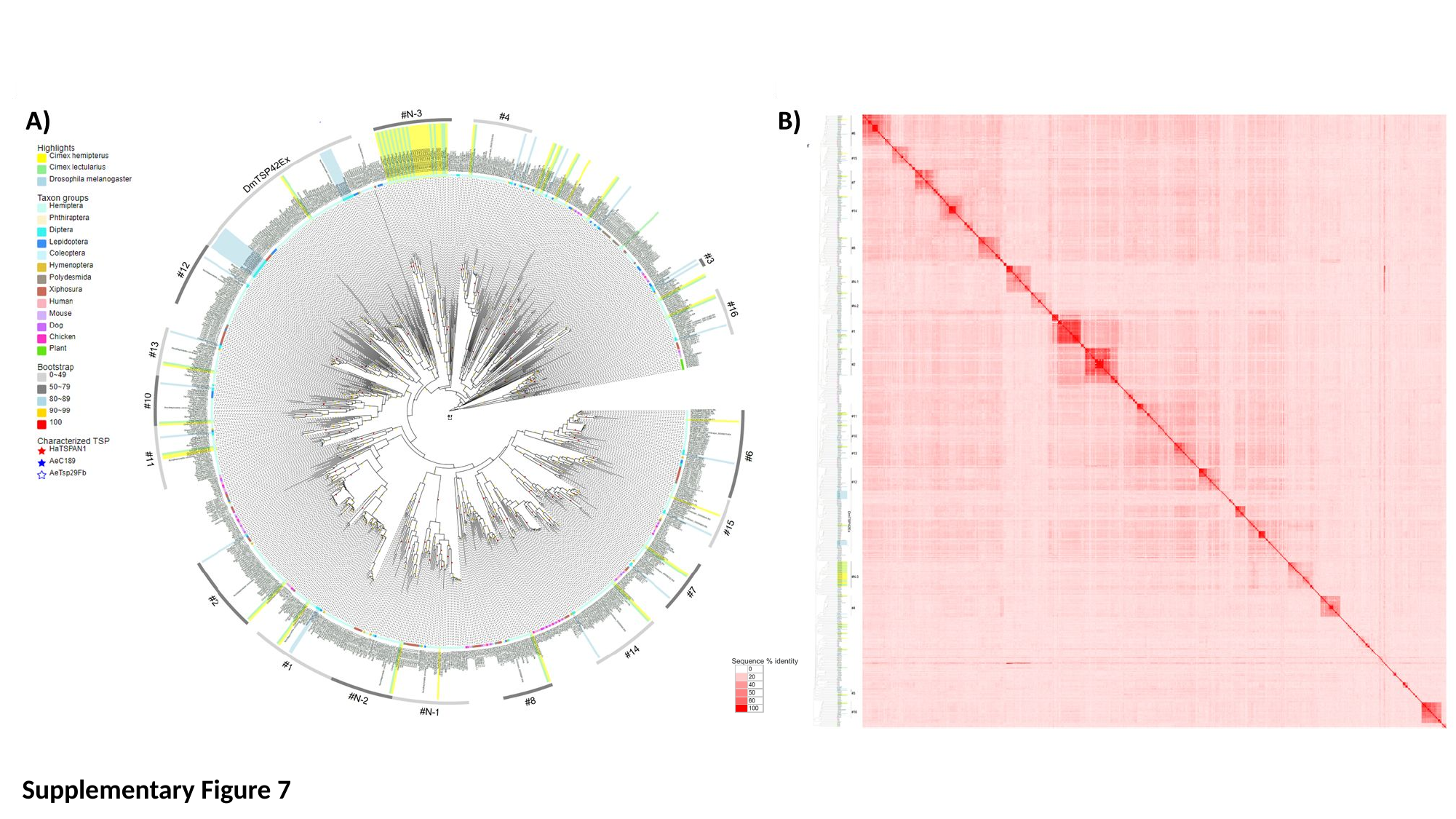

Supplementary Figure 7

## Slide 9
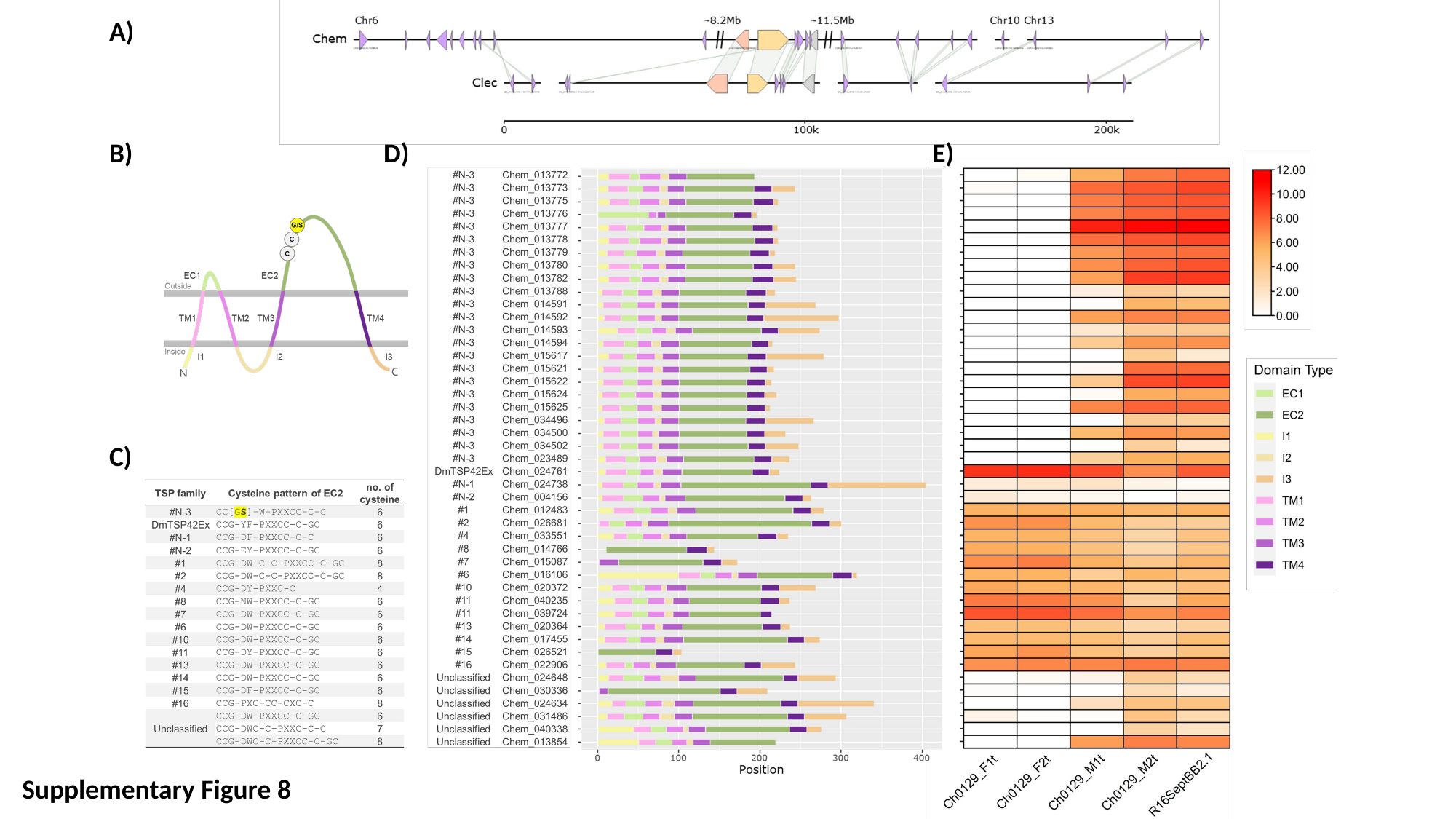

A)
B)
D)
E)
C)
Supplementary Figure 8

## Slide 10
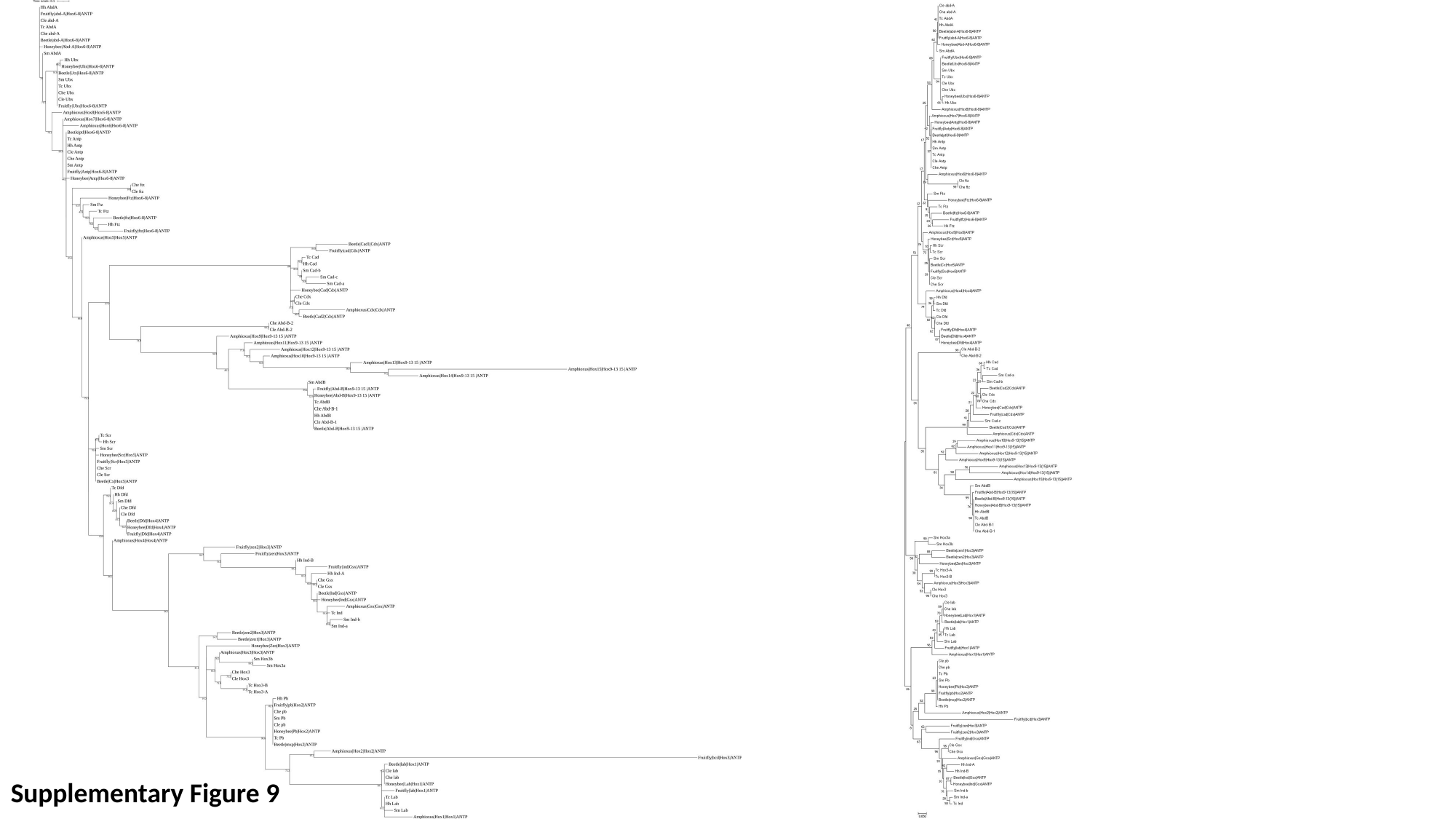

Supplementary Figure 9

## Slide 11
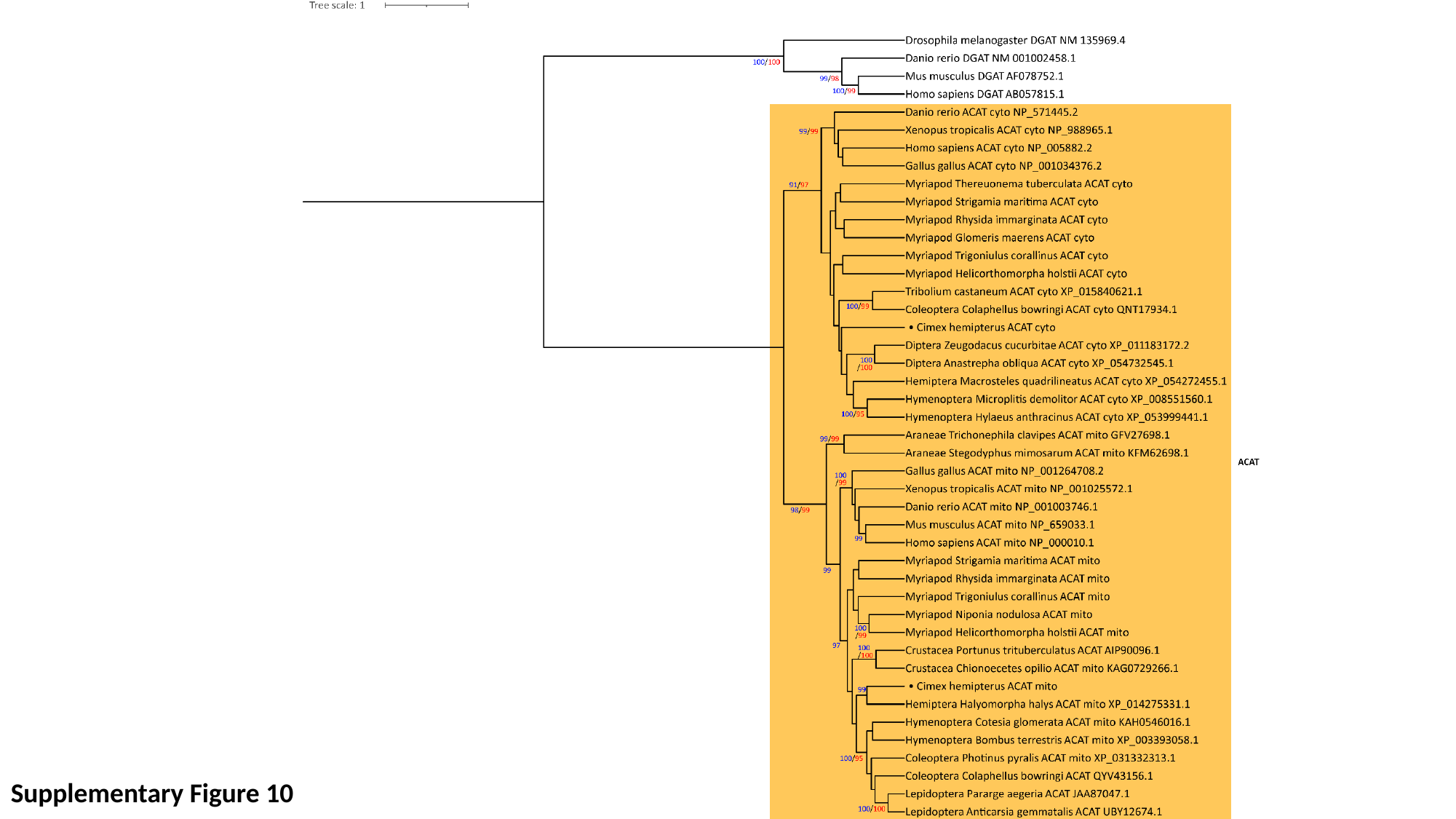

Supplementary Figure 10

## Slide 12
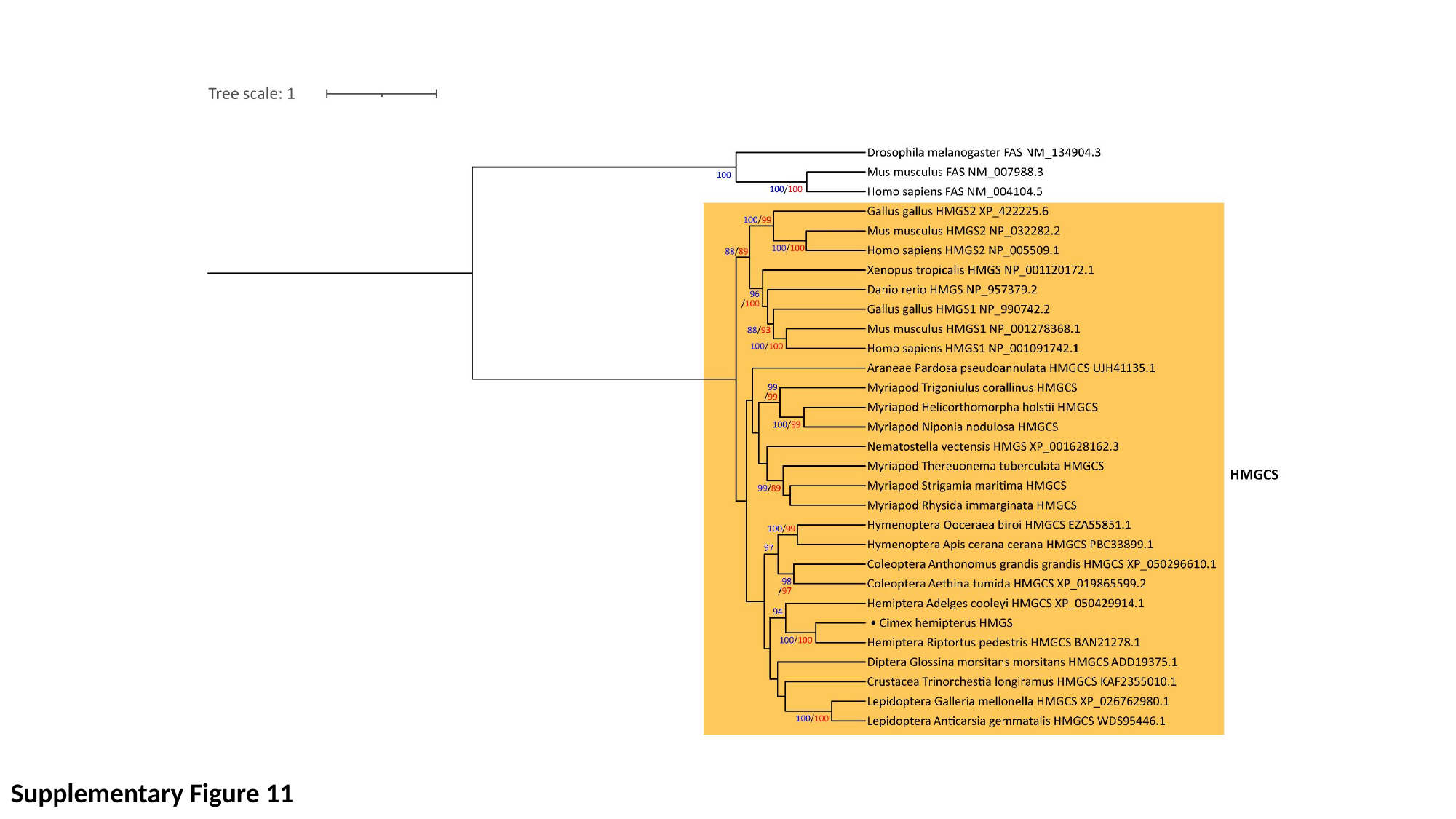

Supplementary Figure 11

## Slide 13
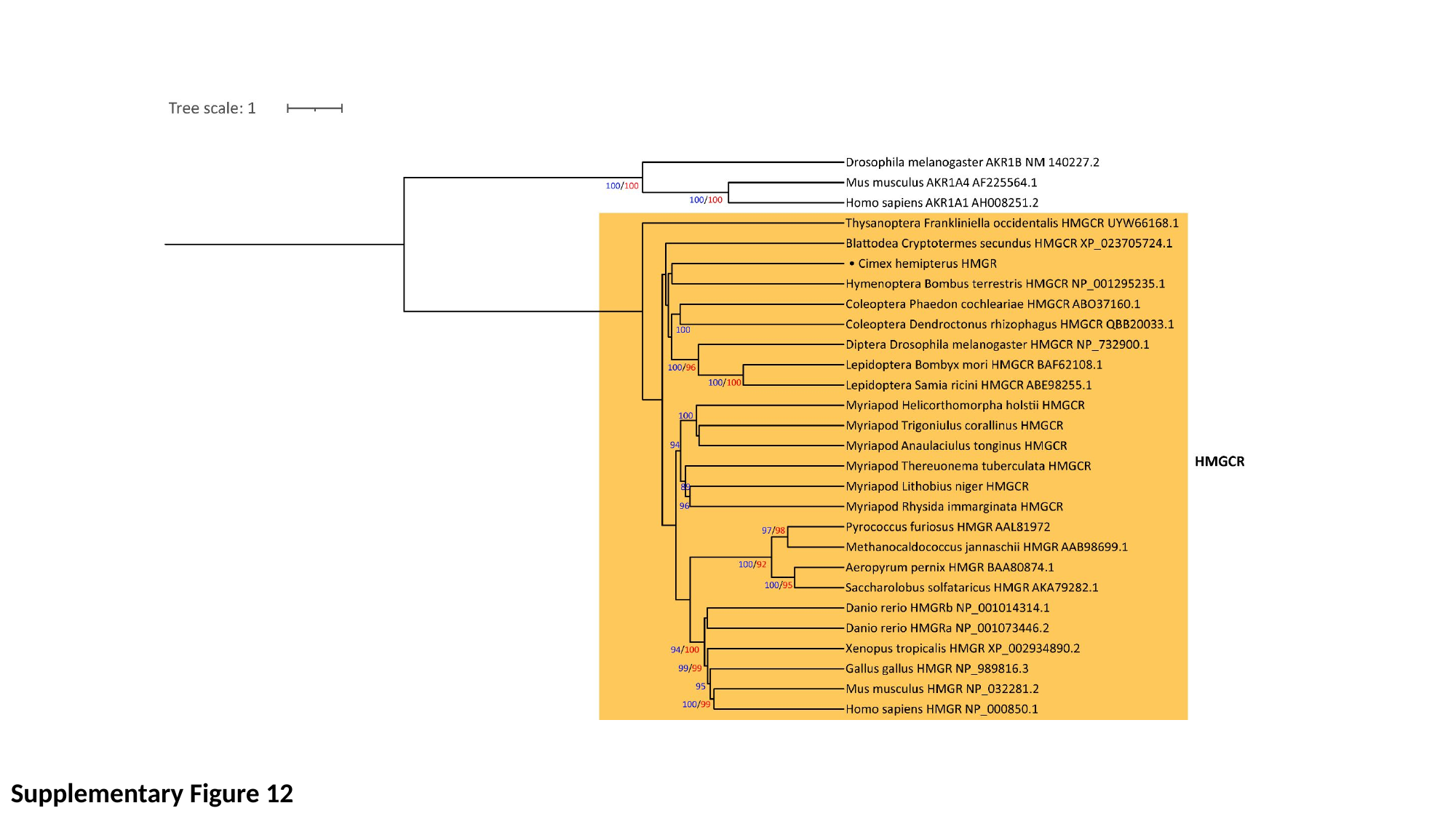

Supplementary Figure 12

## Slide 14
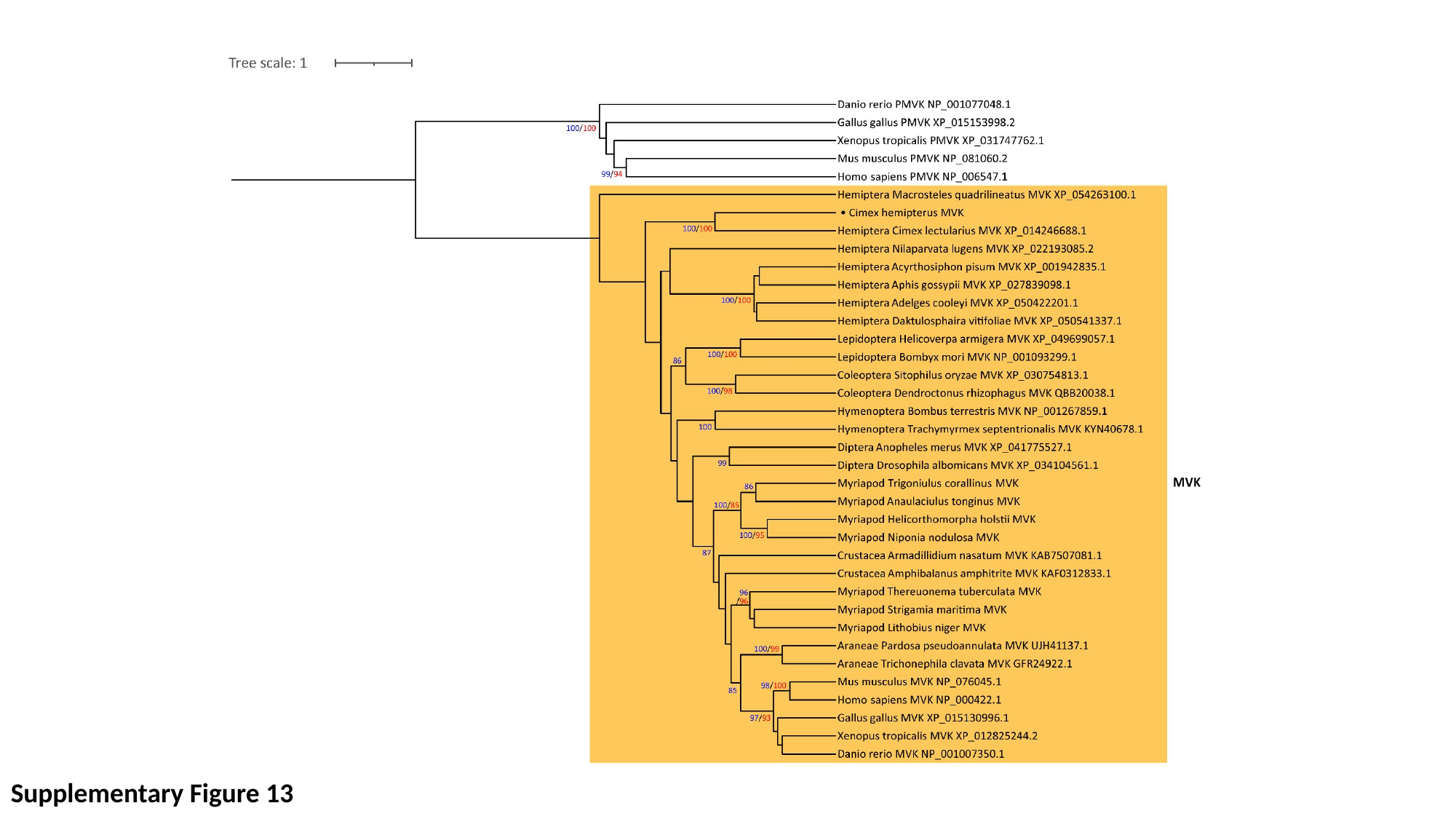

Supplementary Figure 13

## Slide 15
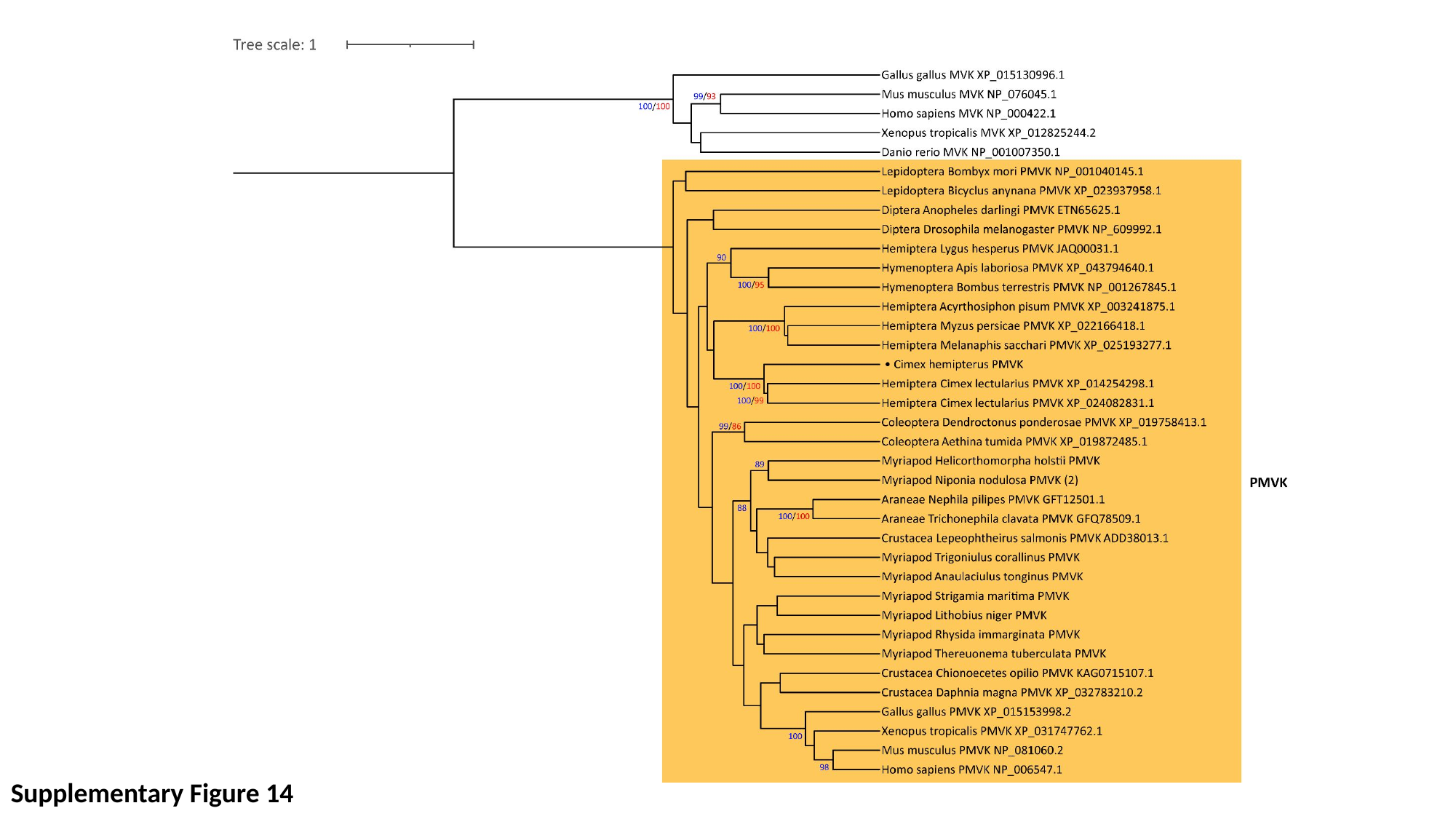

Supplementary Figure 14

## Slide 16
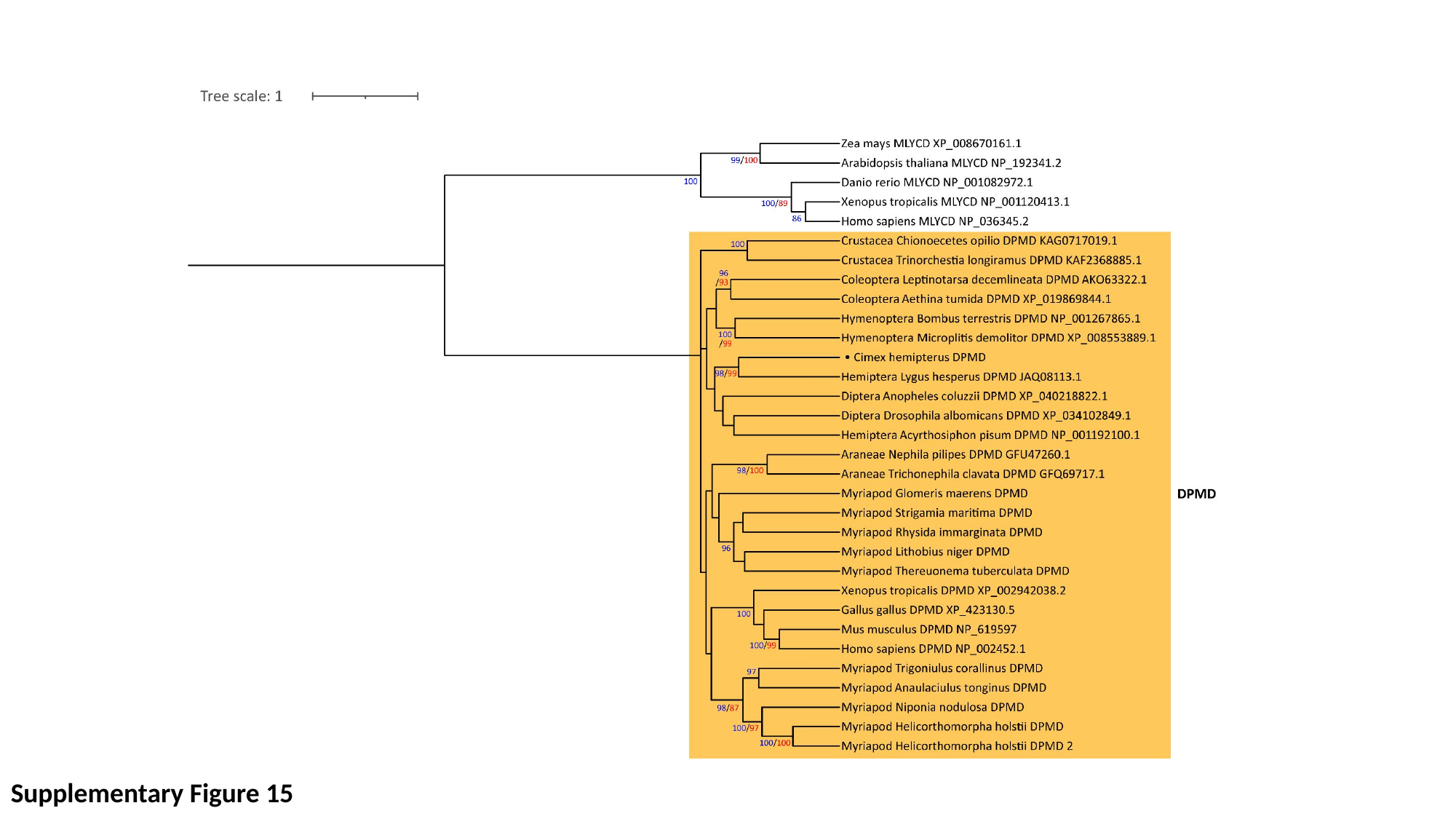

Supplementary Figure 15

## Slide 17
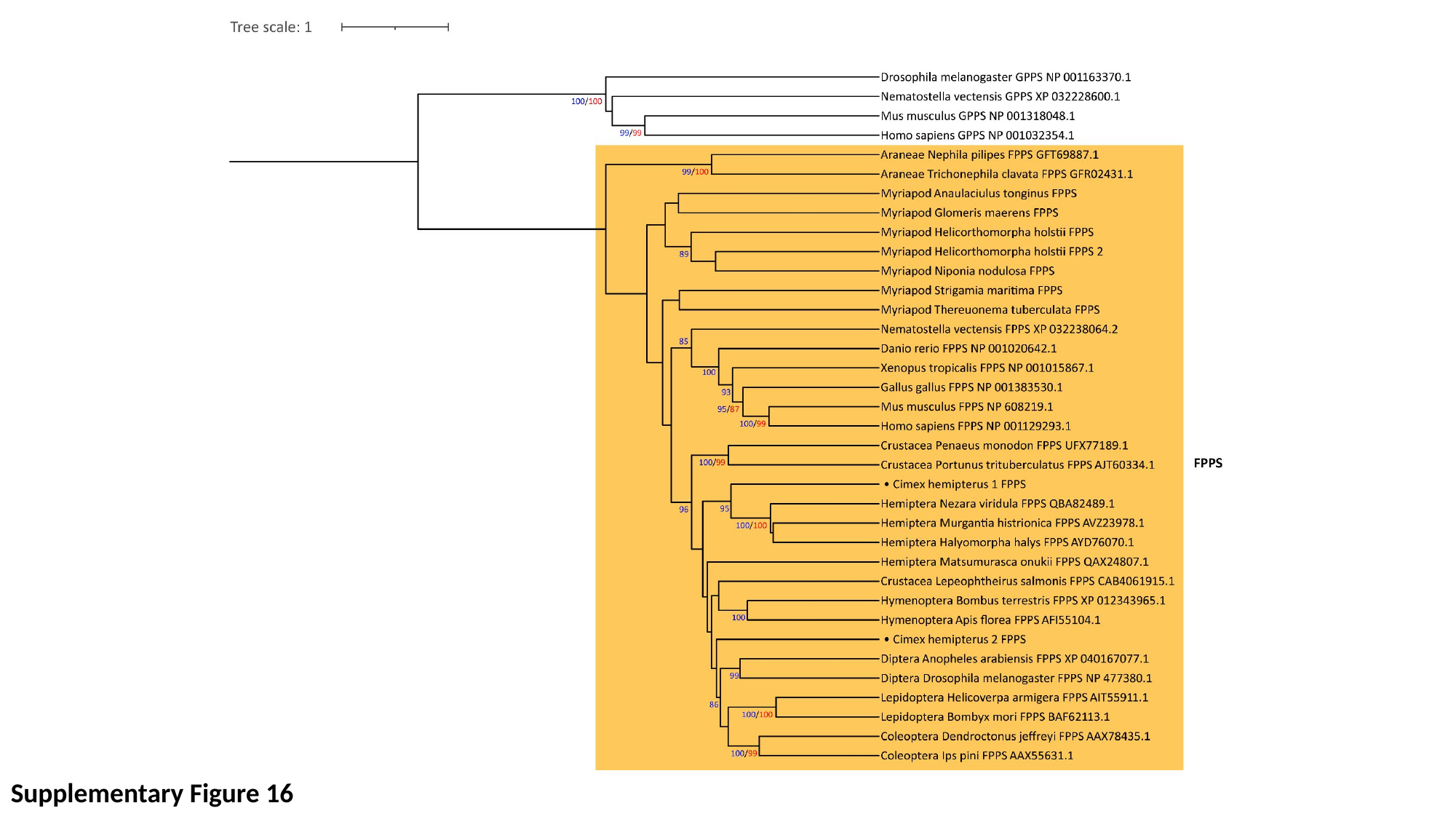

Supplementary Figure 16

## Slide 18
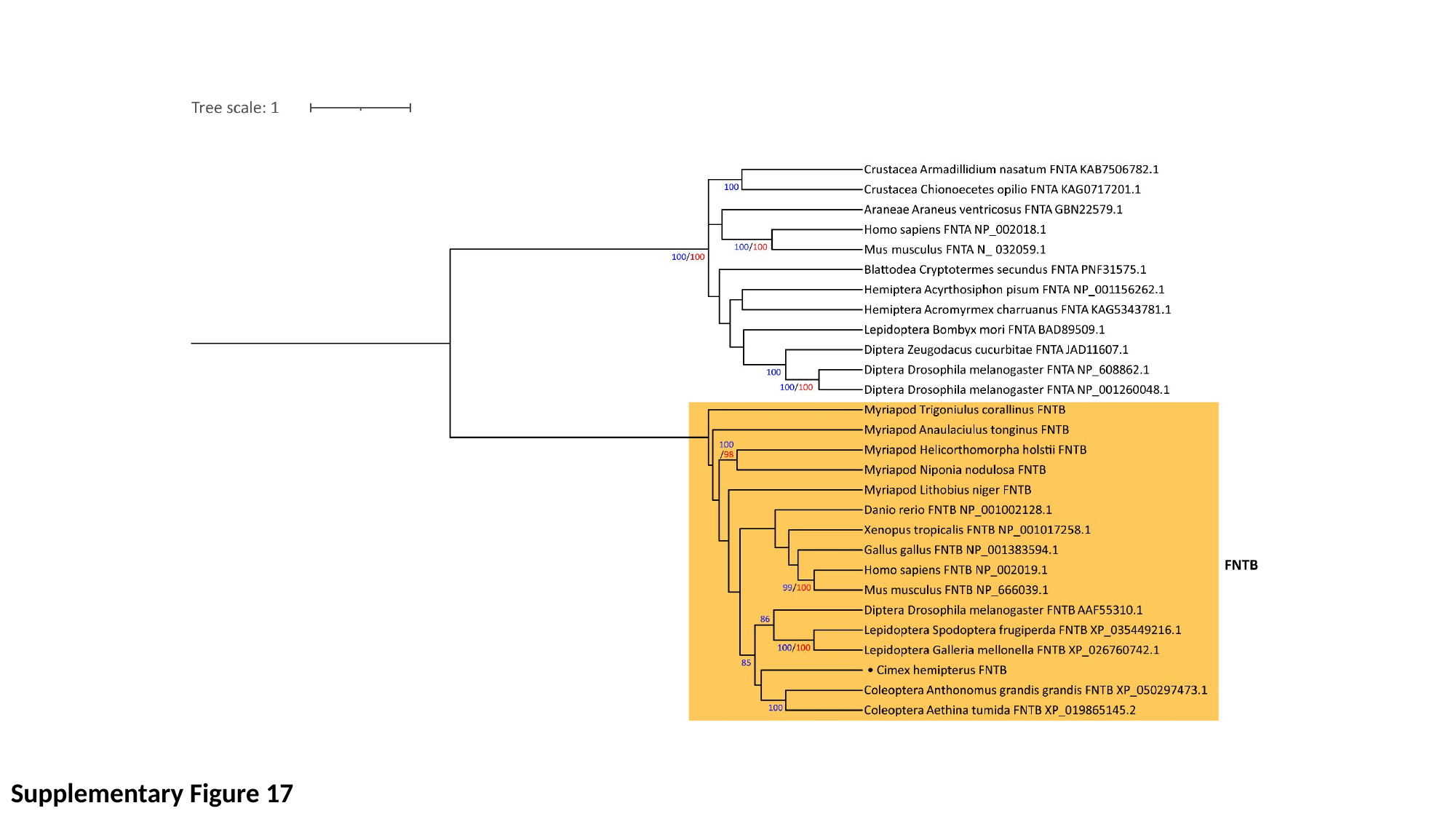

Supplementary Figure 17

## Slide 19
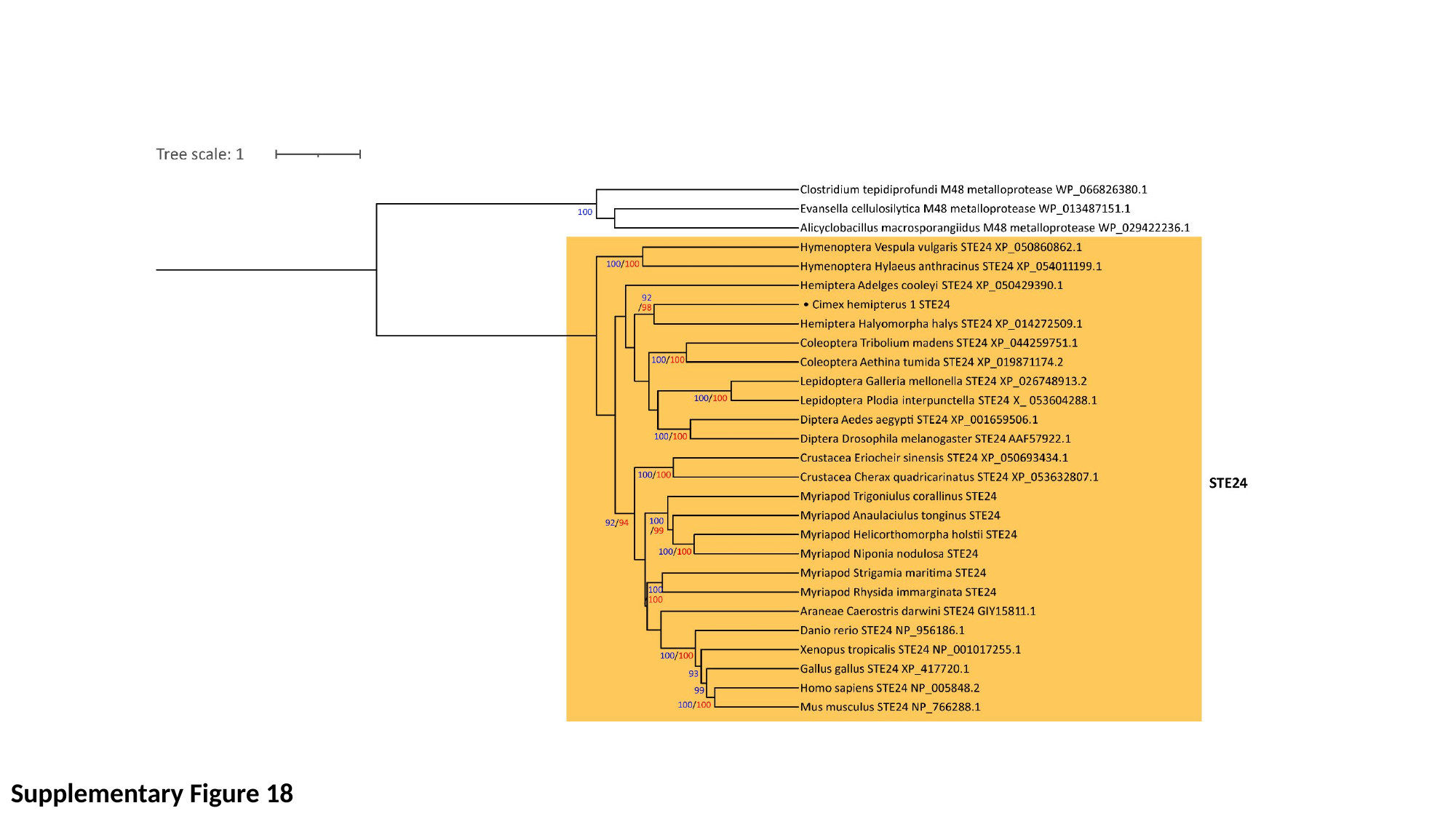

Supplementary Figure 18

## Slide 20
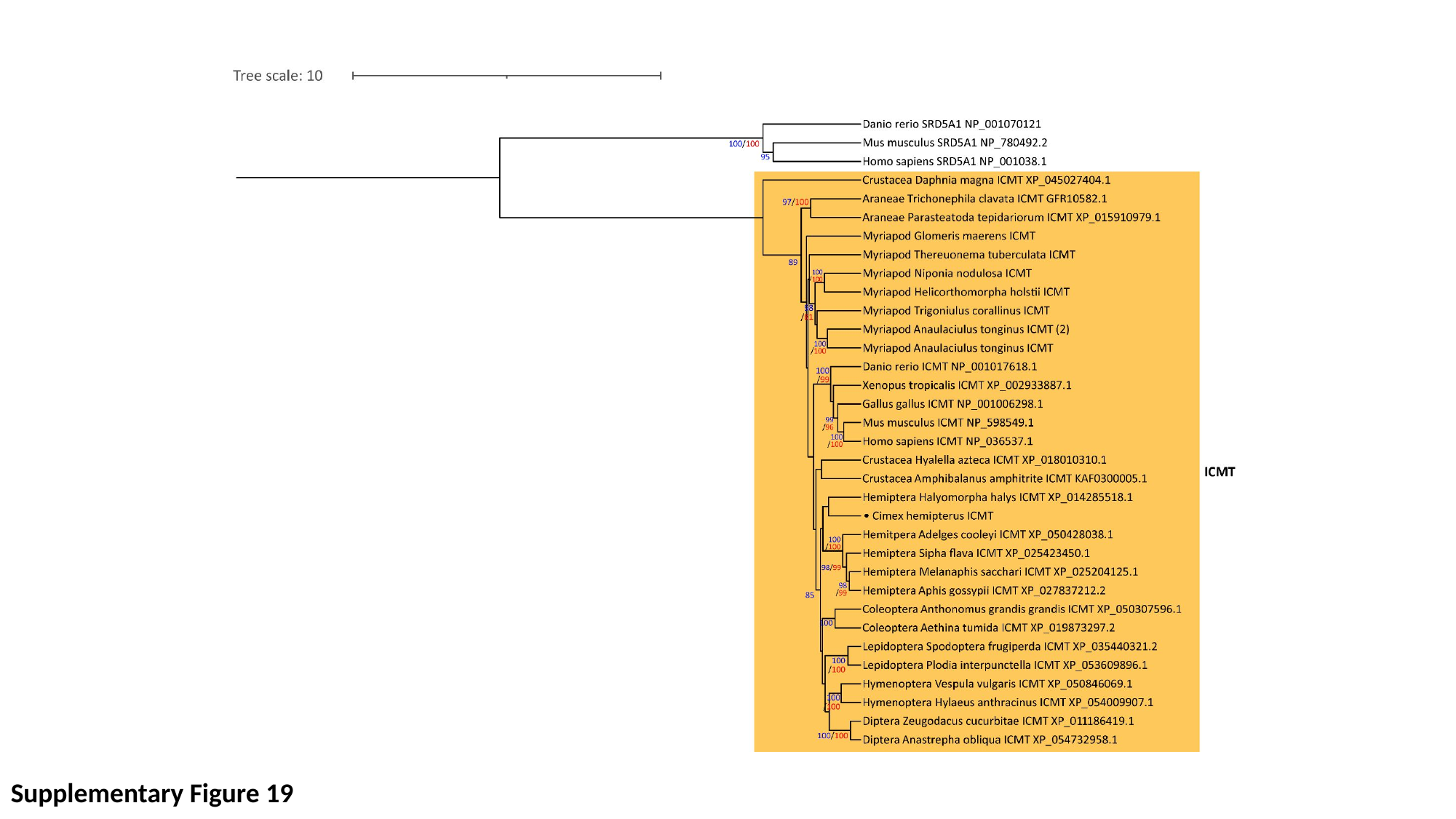

Supplementary Figure 19

## Slide 21
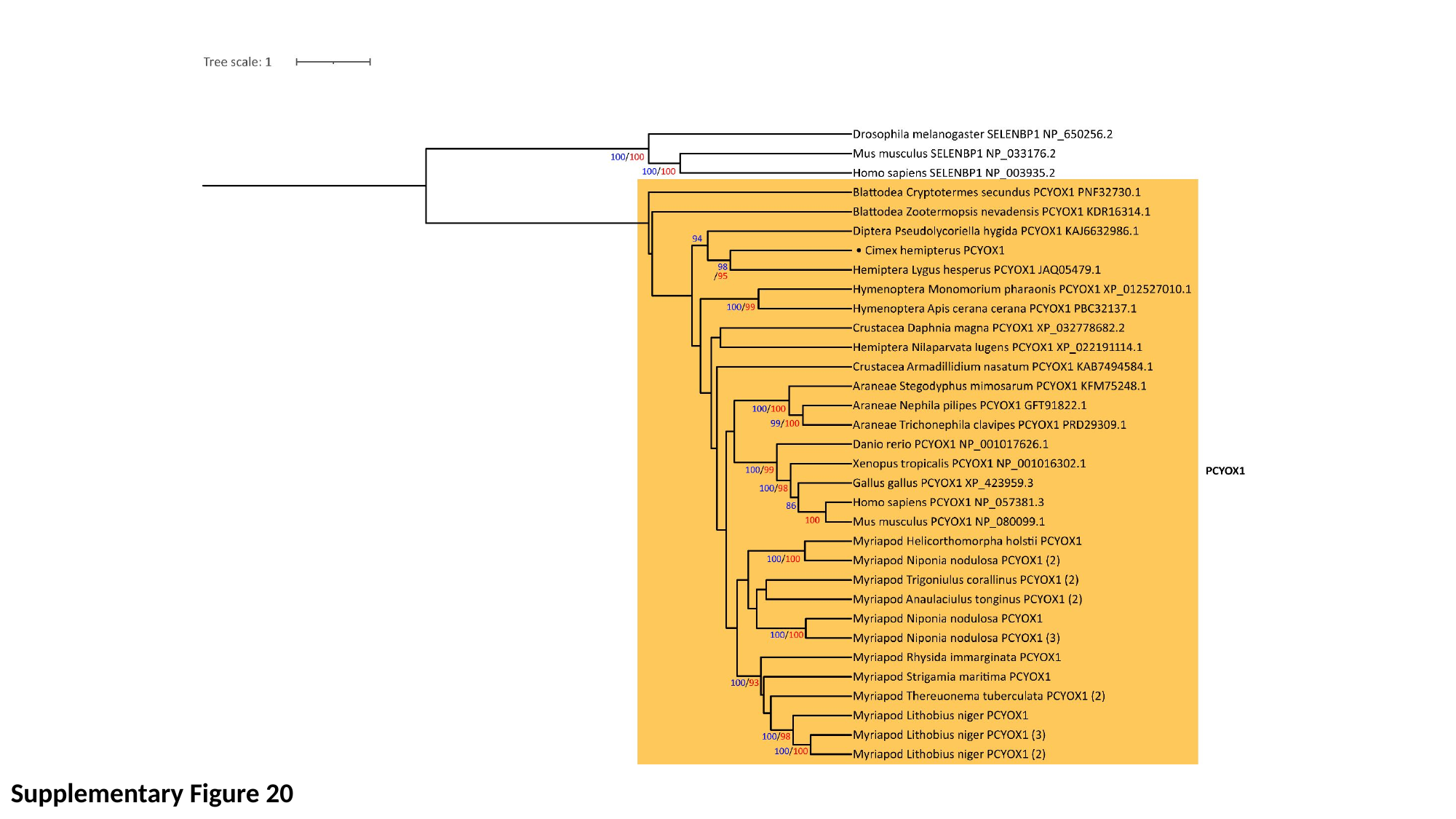

Supplementary Figure 20

## Slide 22
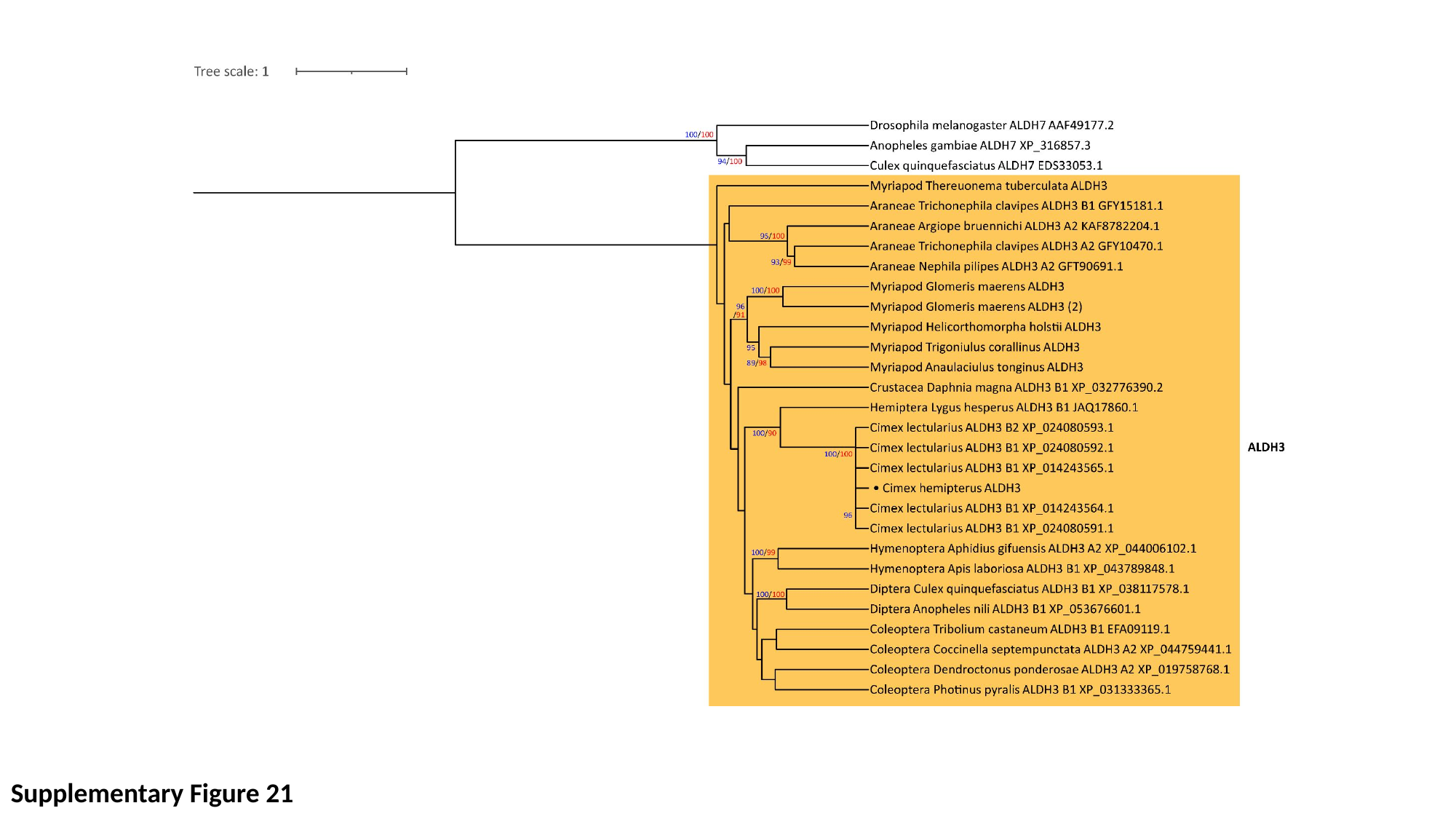

Supplementary Figure 21

## Slide 23
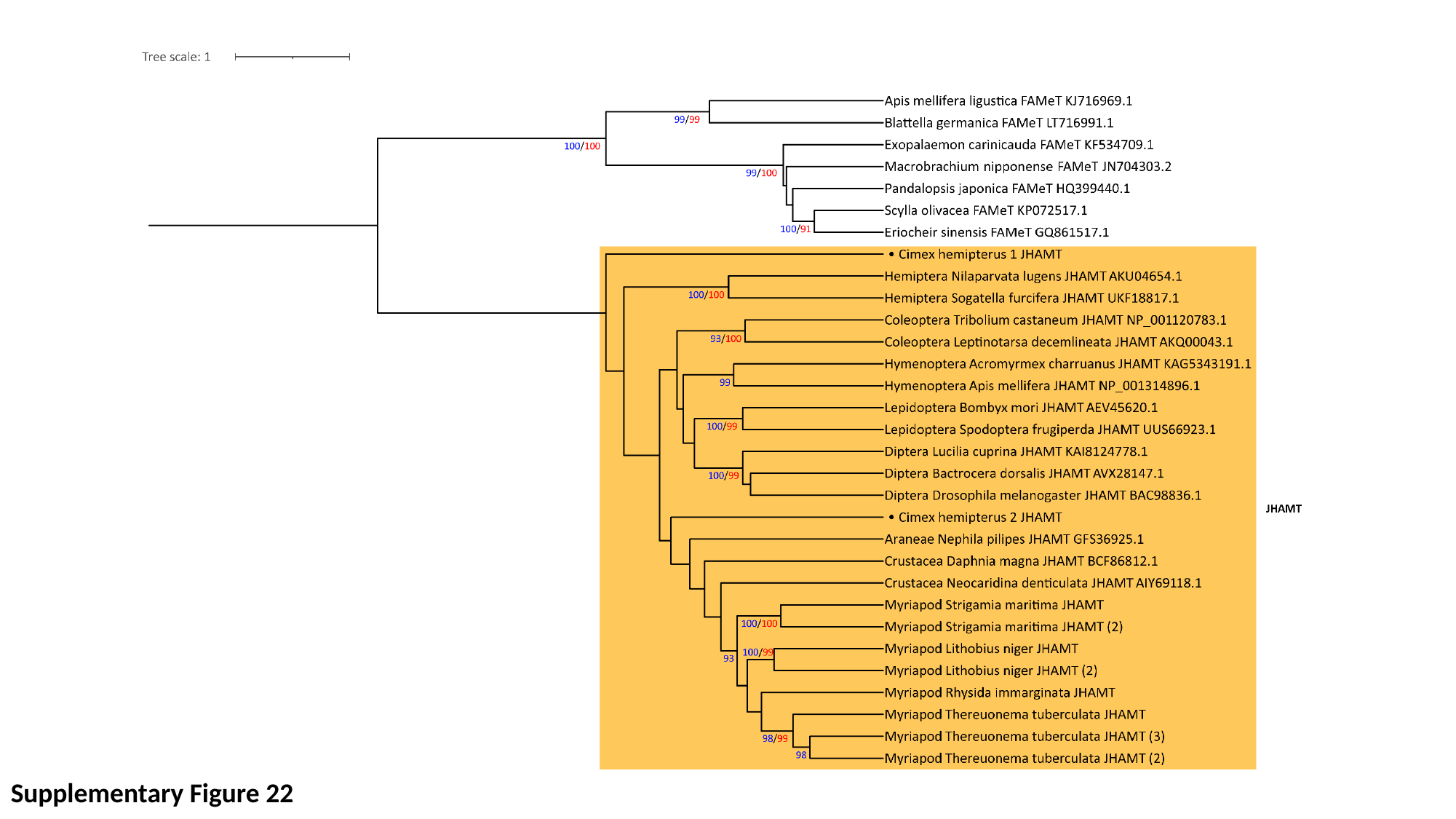

Supplementary Figure 22

## Slide 24
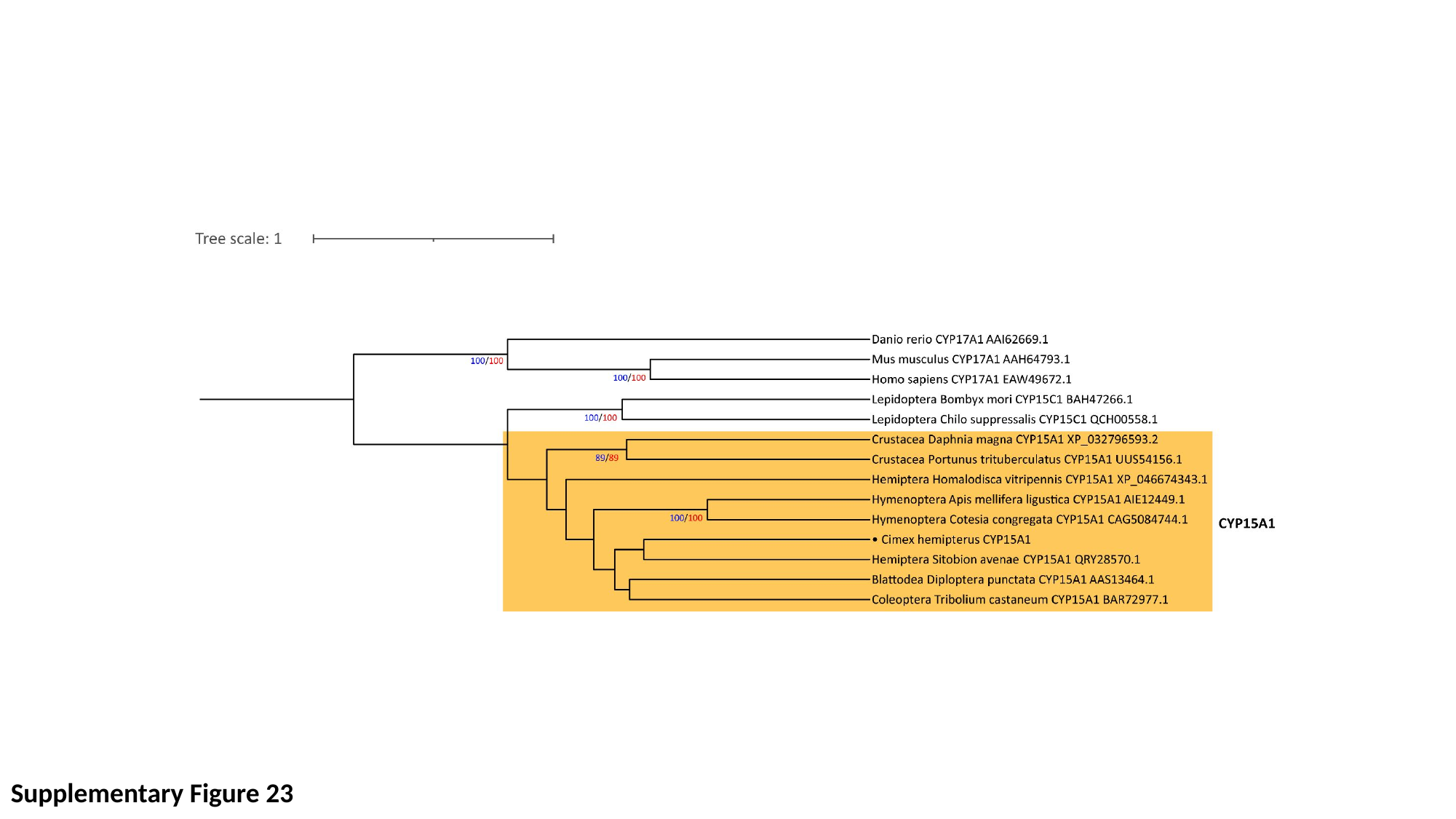

Supplementary Figure 23

## Slide 25
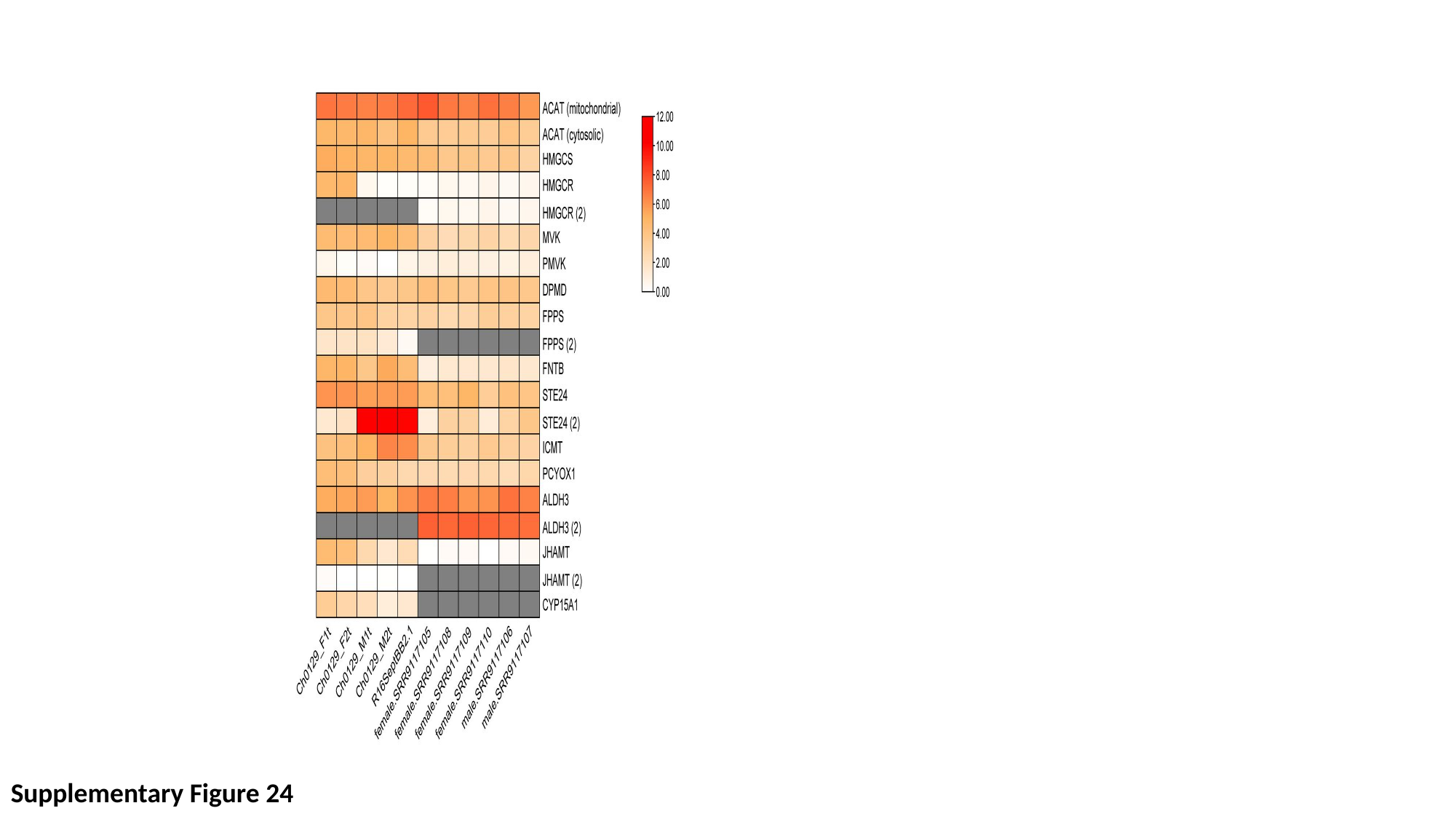

#
Supplementary Figure 24

## Slide 26
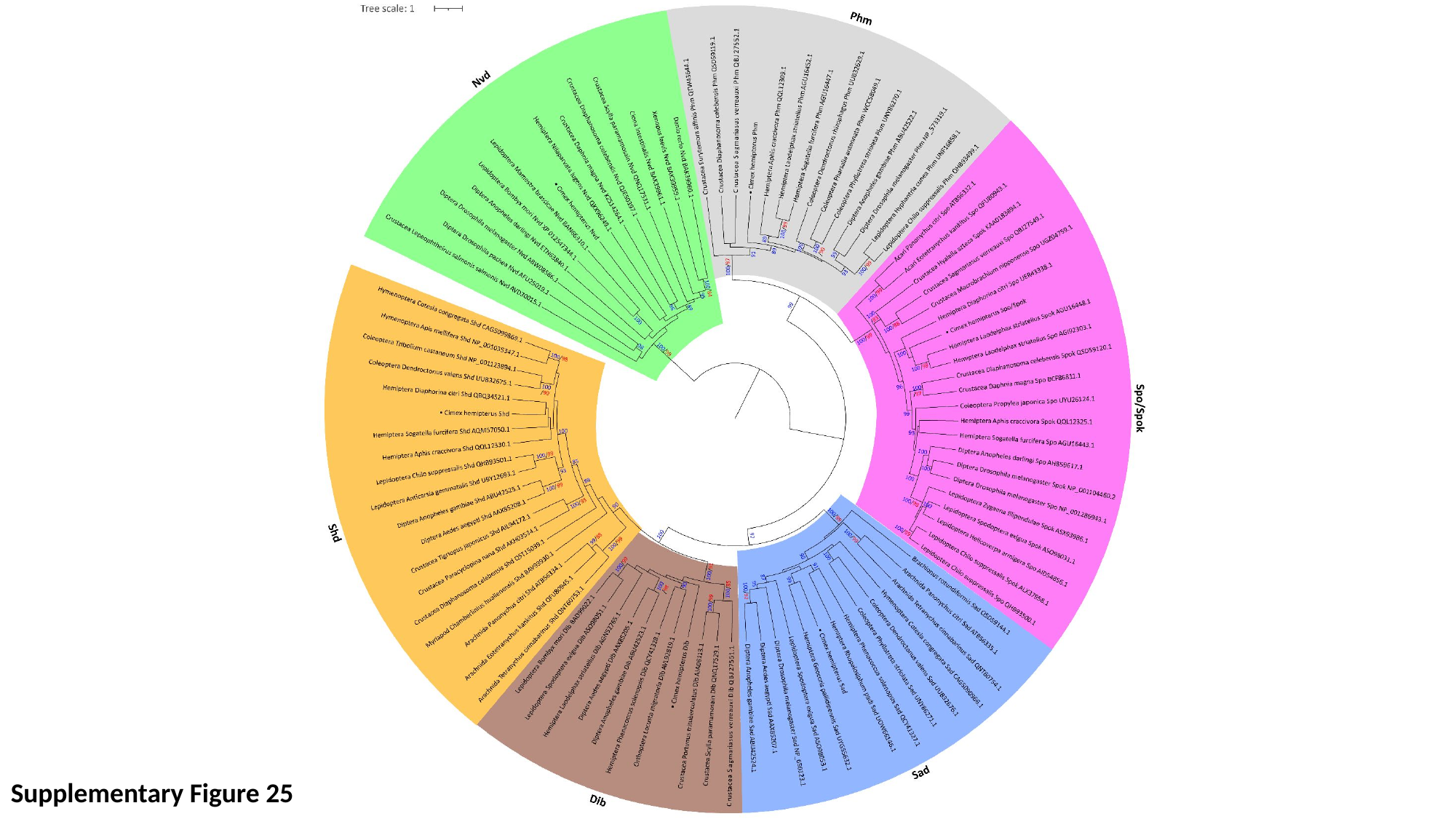

Supplementary Figure 25

## Slide 27
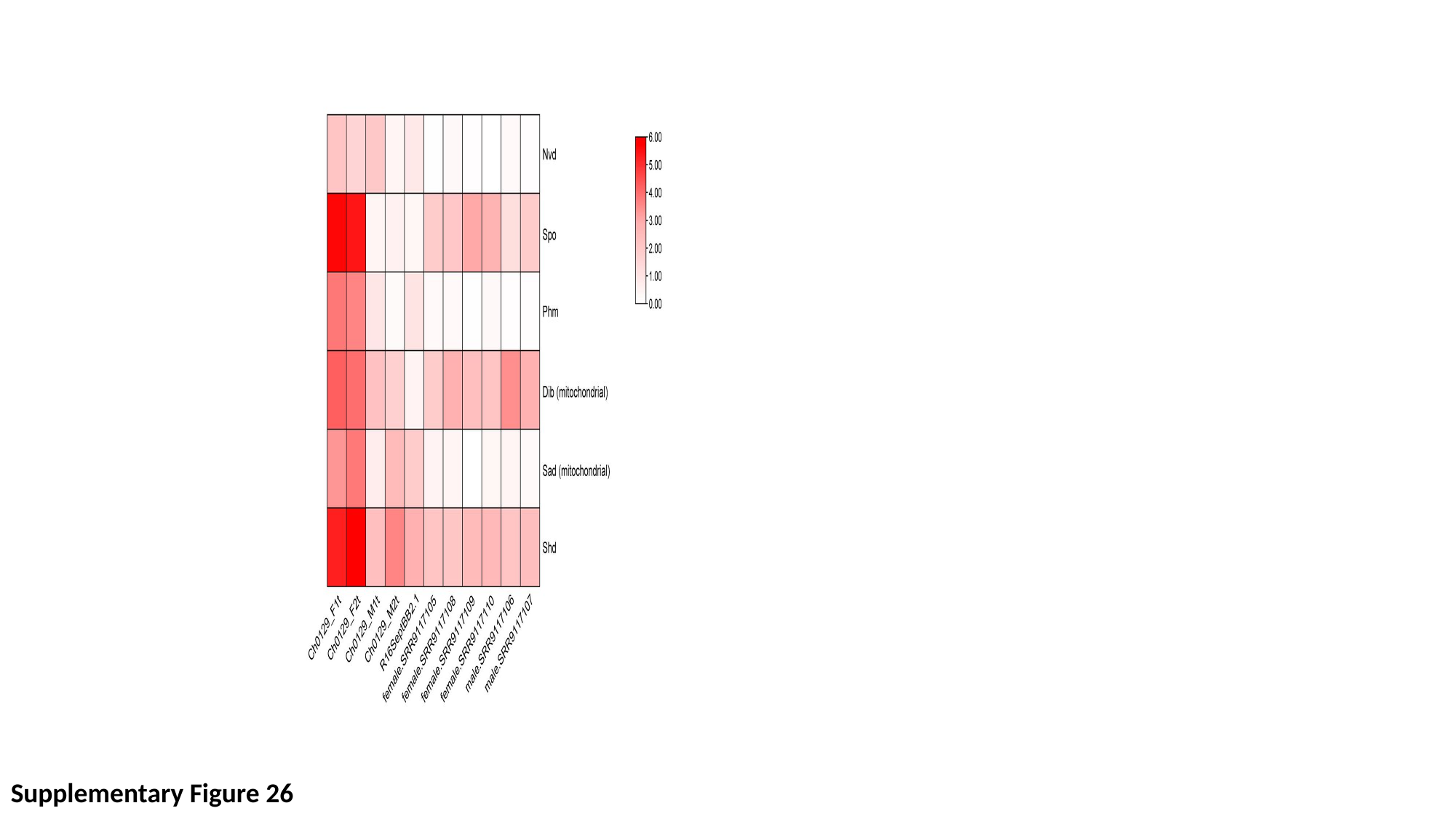

#
Supplementary Figure 26
